# Transcriptomic Alterations in Lung Adenocarcinoma Unveil New Mechanisms Targeted by the *TBX2* Subfamily of Tumor Suppressor Genes

**DOI:** 10.3389/fonc.2018.00482

**Published:** 2018-10-30

**Authors:** Athar Khalil, Batoul Dekmak, Fouad Boulos, Jake Kantrowitz, Avrum Spira, Junya Fujimoto, Humam Kadara, Nehme El-Hachem, Georges Nemer

**Affiliations:** ^1^Departments of Biochemistry and Molecular Genetics, Faculty of Medicine, American University of Beirut, Beirut, Lebanon; ^2^Department of Pathology, Faculty of Medicine, American University of Beirut, Beirut, Lebanon; ^3^Section of Computational Biomedicine, Boston University, Boston, MA, United States; ^4^Department of Translational Molecular Pathology, The University of Texas MD Anderson Cancer Center, Houston, TX, United States; ^5^Division of Cancer Prevention, The University of Texas MD Anderson Cancer Center, Houston, TX, United States; ^6^Faculty of Medicine and Genome Innovation Centre, McGill University, Montreal, QC, Canada

**Keywords:** transcriptomics, T-Box, tumor suppressor gene, demethylation, lung adenocarcinoma

## Abstract

T-box (TBX) transcription factors are evolutionary conserved genes and master transcriptional regulators. In mammals, TBX2 subfamily (TBX2, TBX3, TBX4, and TBX5) genes are expressed in the developing lung bud and tracheae. Our group previously showed that the expression of TBX2 subfamily was significantly high in human normal lungs, but markedly suppressed in lung adenocarcinoma (LUAD). To further elucidate their role in LUAD pathogenesis, we first confirmed abundant expression of protein products of the four members by immunostaining in adult human normal lung tissues. We also found overall suppressed expression of these genes and their corresponding proteins in a panel of human LUAD cell lines. Transient over-expression of each of the genes in human (NCI-H1299), and mouse (MDA-F471) derived lung cancer cells was found to significantly inhibit growth and proliferation as well as induce apoptosis. Genome-wide transcriptomic analyses on NCI-H1299 cells, overexpressing TBX2 gene subfamily, unraveled novel regulatory pathways. These included, among others, inhibition of cell cycle progression but more importantly activation of the histone demethylase pathway. When using a pattern-matching algorithm, we showed that TBX's overexpression mimic molecular signatures from azacitidine treated NCI-H1299 cells which in turn are inversely correlated to expression profiles of both human and murine lung tumors relative to matched normal lung. In conclusion, we showed that the TBX2 subfamily genes play a critical tumor suppressor role in lung cancer pathogenesis through regulating its methylating pattern, making them putative candidates for epigenetic therapy in LUAD.

## Introduction

Lung cancer remains the most commonly diagnosed cancers and the leading cause of mortality among all cancers (1–3). Histologically, it is divided into two main subtypes: small cell lung carcinoma (SCLC), and non-small-cell lung carcinoma (NSCLC). NSCLC is further classified into three types with lung adenocarcinoma (LUAD) representing around 40% of the cases. This type is characterized by an aggressive phenotype; and as such is considered to be a fatal tumor type with an overall prognosis of <5 years (4–6). This high rate of mortality among LUAD patients is largely due to the lack of early detection strategies and the failure of advanced targeted therapies. In addition, the many adverse side effects of the current treatment regimens underscore the valuable merits of identifying novel mechanisms regulating NSCLC progression as new potential therapeutic targets in LUAD. An example of such mechanisms is the discovery that epigenomic alterations contribute significantly to the early onset of lung cancer and its etiology (7–9). Since epigenetic modifications are reversible, many ongoing clinical trials are underway using novel molecules that would specifically target these mechanisms taking into account that as such they will affect cancer progression by suppressing proliferation and invasion (10).

T-box proteins are an evolutionary conserved family of transcription factors (11) that exhibits a lineage-specific role during embryogenesis by enhancing and/or repressing the transcriptional expression of a network of genes (12). TBX family members are sub-classified according to their sequence homology and pattern of expression (13, 14). Amongst these classes, the TBX2 subfamily made of *Tbx*2,3,4, and 5 that has been extensively studied because of the specific and complementary roles of its pairs (*Tbx2*-*Tbx3*) and (*Tbx4*-*Tbx5*) in shaping up limb identity, and cardiac chamber specification in different organisms (15, 16). The 4 members were shown to be particularly expressed early on in the lungs of embryonic mice, when the lung bud starts developing and in the developing lungs (lung buds and trachea) (17, 18). In gene targeting studies, *Tbx2* was shown to restrict cell proliferation and inhibit lung mesenchyme differentiation for control of lung growth (19, 20). Depletion of Tbx4 or Tbx5 was shown to impede bronchial differentiation (21). Besides their critical role during embryonic development as demonstrated by gain and loss of function *in vitro* and *in vivo* assays across the species, the aberrant expression of these genes was also associated with several neoplasms, including melanomas, breast, and pancreatic cancer (12, 22). In most of these cancer types, members of the TBX2 family have different patterns of expression, mainly they are unaffected, or overexpressed, but rarely repressed (23–25). In contrast, we have recently shown a unique common suppression pattern in LUADs by the analysis of different large cohorts of patients (26). Our results are supported by data from The Cancer Genome Atlas (TCGA) with its 483 LUAD tumors and 59 controls. This marked suppression was further detected at the pre-malignancy stage, and even in the normal-appearing airway cancerization field in NSCLC. In addition, using a dataset comprised of 164 suspect smokers, we showed that the suppression of these factors was found to be a significant predictor of lung cancer in the suspect high-risk smokers (*p* < 10^−5^) (26).

To further elucidate the role of the TBX2 subfamily in human LUAD pathogenesis, we opted to assess the effects of their re-expression in a lung cancer cell lines. We first confirmed the marked downregulation of *Tbx2-5* transcripts in the various lung cancer cell lines as compared to normal lung tissues. We then assessed the transient independent overexpression in human NCI-H1299 and mouse MDA-F471 cells for each gene. The observed dramatic effect on cell growth and proliferation was quantified and validated. This was further dissected at the molecular level by analyzing and cross-contrasting the transcriptome profile of these cells as compared to the control by RNA sequencing. We found only a shared pathway activated by all four members, and many other suppressed pathways. The activated demethylation pathway was paralleled to a suppressed methylation process, both affecting the epigenetic signature of lung cancer cells. Interestingly, the expression pattern induced by *TBX2* subfamily correlated with gene signatures from NCI-H1299 treated with demethylases inhibitors. Finally, we showed that LUAD gene signatures from both mouse tumor model and public datasets were anti-correlated with transcriptional signatures from cells overexpressing TBX2-5.

## Materials and methods

### Immunohistochemistry

Consecutive sections (8 μm) were prepared from formalin-fixed paraffin embedded normal human lung tissues. Slides were dewaxed, rehydrated with graded alcohol, then dipped in 3% hydrogen peroxide. Antigen retrieval was carried out in citrate buffer (pH 6.0) in a microwave, followed by a 1 h blocking in 3% BSA (Amresco Life science, Cat#0332-100G) in 0.2% PBT (PBS 1X with 0.2% Tween-20). Primary rabbit-antibody against human TBX2 (Ab33298), TBX3(Ab99307), TBX4(Ab28634), TBX5(Ab23665) and as a negative control rabbit IgG was used (Ab27478) with a dilution of 1:100 were applied to the sections, respectively, and incubated overnight at 4°C. Subsequently, slides were incubated with 1:250 Biotinylated species-specific secondary antibody (Cat# RPN1004V, GE healthcare UK limited) followed by 1:250 streptavidin–peroxidase conjugate, and antibody-specific binding was visualized with 3,3-diaminobenzidine solution (DAB–Sigma, Cat#D3939-1SET). Lastly, slides were counterstained with Methyl Green and mounted.

### Cell lines and transfection assays

Human lung cancer cell lines (NCI-H3255, NCI-H1299, NCI-H1792, and NCI-H1944) and mouse lung adenocarcinoma cells (MDA-F471) derived from 4-(methylnitrosamino)-1-(3-pyridyl)-1-butanone (NNK) exposed *Gprc5a*-knockout female lungs were obtained from Dr. Humam Kadara. All the above cells were grown in DMEM-F12 low glucose medium supplemented with Fetal Bovine Serum (FBS-Sigma, Cat#F9665), 1% Penicillin/ Streptomycin (Biowest-Cat#L0022-100). Normal Human Bronchial Epithelial Cells (NHBE) and immortalized human bronchial epithelial cells, HBEC1 and HBEC2 were cultured in growth factor-supplemented medium (BEGM, Lonza). HEK293 cells (Human Embryonic Kidney cells) were cultured and maintained in Dulbecco's Modified Eagle Media (DMEM-Sigma, Cat#D0819) supplemented with 10% Fetal Bovine Serum (FBS-Sigma, Cat#F9665), 1% Penicillin/ Streptomycin (Biowest-Cat#L0022-100) and 1% sodium pyruvate (Sigma-Cat#S8636). Incubation of cells was carried out in a 5% CO2 humid atmosphere at 37°C. Mouse HA tagged pCGN-*Tbx5* and human HA tagged pCGN-*TBX3* were previously described while human Flag tagged pORF-TBX (2 and 4) were purchased from Origene. NCI-H1299 and F471 cells were transfected with the expression vectors using Lipofectamine 2000 (Life Technologies) according to the manufacturer's instructions with an efficiency of ~45%. Transfection was done after 1-h incubation with Penicillin/Streptomycin free cell culture medium. HEK293 cells were transiently transfected using Polyethylenimine (Sigma) as previously described (27).

### Total RNA isolation

Total RNA was isolated using the Invitrogen Life Technologies TRIzol reagent according to the manufacturer's protocol and quantified using the Nanodrop 1,000 spectrophotometer (Thermo Scientific) at the Molecular Core Facility at AUB. Briefly, 70% confluent cells seeded in 10 cm^2^ petri dishes were scraped and centrifuged for 15 min at 13,000 rpm at 4°C. RNA quality was assessed based on RNA integrity numbers (RINs) using a 2,100 Bioanalyzer (Macrogen, https://dna.macrogen.com/eng/). For lung tissues, eight 10 μm slices were deparaffinized using 100% xylene, and total RNA extraction was done using RecoverAll™ Total Nucleic Acid Isolation Kit (Life Technologies) according to manufacturer's instruction.

### RNA-seq analysis

Resulting pairs of FASTQ generated from Macrogen Illumina pipeline (paired-end sequencing), for each of the settings (Controls, *TBX2, TBX3, TBX4, TBX5*), were aligned to the Human reference genome build GRCh37.75 using STAR v2.3.0. Mapped reads were quantified using feature Counts v1.5.4 using default settings, and corresponding GTF gene annotation file was used to map reads to annotated transcripts. Genes with raw counts above 5 in more than half of the samples (*N* = 18) were kept for further analysis. Differential expression analysis (DEG) to identify significant gene regulation, in TBX-transfected cells with respect to control NCI-H1299, was performed using DESeq2.

### *In silico* pathway analysis

Genes were assigned to Reactome pathways and gene sets of sizes between 15 and 250 genes were retained for gene set enrichment analysis (GSEA). Genes were ranked with respect to the t-stat from DESeq2 output. We created a GSEA output matrix with pathway enrichment scores (NES) in rows and all four contrasts (*TBX2* vs. control, *TBX3* vs. control, *TBX4* vs. control, *TBX5* vs. control) in columns. We used custom scripts in R to remove highly overlapping Reactome pathways. Consistent pathways across all four contrasts were kept for further validation.

### Assessment of Tbx-induced gene signature in publicly available human and murine non-small cell lung cancer datasets

From each of the four comparisons described in previous sections, we selected the first 75 genes at the top (most up-regulated DEG) and the last 75 genes at the bottom of the list (most down-regulated DEG) after sorting each ranked list of genes, from DESeq2, by the t-statistics. This was denoted as the query gene signature. Raw microarray public data have been extracted from hypomethylating treatment instances (azacitidine and decitabine) in non-small cell lung cancer cell line NCI-H1299 (28) or from three clinical non-small cell lung cancer cohorts GSE43458, GSE27262, and GSE10072 (29–32). For each dataset, differentially expressed genes between treated and untreated, or tumors and controls were calculated with the limma package in Bioconductor. Then, a connectivity score (range = [−1, +1]) is computed between the query signature and the respective profiles from GEO (33). For example, a “negative connectivity” suggests that Tbxs-induced signature pushes toward a “normal lung phenotype.” In contrast, a “positive connectivity” suggests that one signature mimic the other. Similar analysis was performed on ranked gene lists from the GSE107774 expression dataset (34)comprising three adenocarcinomas, three adenomas and five matched normal lung tissues from tobacco carcinogen exposed *Gprc5a*^−/−^ mice (35, 36).

### Protein extraction and western blots

Nuclear proteins were extracted as previously described (37) and resolved on a 10% SDS-PAGE. The protein samples were boiled for 10 min and run on denaturing SDS-PAGE for about 1.5 h then transferred to a PVDF membrane (Cat#10600023, Amersham, UK). The membrane was blocked in 5%TBT (TBS-0.02% Tween 20) skimmed dry milk for 45 min at RT. The membrane was incubated with primary antibodies (anti-Flag or anti-HA) or anti-TBX (2,3,4,5) and anti-*GAPDH* or anti-β*Actin* overnight at 4°C. On the 2nd day, the membrane was washed three times with TBT (TBS 1X with 0.2% Tween 20) and incubated with secondary anti-mouse or anti-rabbit-HRP (1:50,000) for 1 h at RT. Development was done using ECL™ Western Blotting Detection Reagents (Amersham, GE healthcare, Cat# RPN2106). The protein bands were visualized using the Chemidoc MP imaging system (Bio-Rad) and quantified using Image J software.

### Immunofluorescence

NCI-H1299 cells were plated onto 12-well Costar culture plates on coverslips at sub-confluency ~60% per well. Transient transfection was done on the 2nd day of the seeding using 2 μl Lipofectamine 2000 (Life Technologies) and a total of 2 μg of DNA per well. Cells were fixed 24 h post-transfection using 4% paraformaldehyde/PBS for 20 min and washed again with PBS. The cells were then blocked with 3% BSA/PBS solution for 1 h. followed by two primary antibodies (rabbit anti-HA or mouse anti-Flag and anti-mouse Ki-67 or rabbit Ki-67, respectively) diluted (1:300) in BSA/PBT and added to the cells with an overnight incubation at 4°C. The cells were then washed with PBT 3 times, before adding the secondary antibody (donkey anti-rabbit IgG biotinylated, RPN1004V, or anti-mouse IgG biotinylated, GE Healthcare) with a dilution of 1:250 in BSA/PBT for 1 h at RT with shaking. After washing 3 times with PBT, cells were incubated with ChromeoTM 488 Streptavidin (Santa Cruz), and Alexa Fluor 555 for 1 h at RT with shaking. Nuclei staining was also performed by applying Hoechst, diluted 1:30 in water, for 30 min. The cells were then washed with PBS and mounted on a rectangular slide containing an anti-fading agent DABCO (Sigma–Aldrich). The slides were examined using the Olympus BH-2 microscope at the molecular core facility at AUB.

### Trypan blue exclusion assay and cell cycle analysis

Cells were seeded in duplicate wells for each experimental condition at a density of 2.5 × 10^4^ cells/well in 12-well plates, and then transfected the following day with the specific overexpression vector. 24 and 48 h following transfection, cells were washed with 1x PBS, trypsinized, and mixed with 0.4% Trypan blue solution in a 1:1 ratio (Sigma Aldrich) then counted under a light microscope using a hemocytometer. For cell cycle analysis, cells were plated in 12 well plates (2.5 × 10^5^ cells/well) and transfected after 24 h with the assigned plasmids or the corresponding empty vector. Cells (including floating cells in the medium) were harvested using trypsin at the indicated times, washed twice with ice cold PBS and fixed with pre-chilled 70% ethanol at 4°C for at least 48 h. The cells were then pelleted by centrifugation at 1,500 rpm, washed with PBS and resuspended in 150 μl of PBS and digested with 100 μl of RNAse A (50 μg/ml). Staining was done with 25 μl PI solution at 4°C in the dark for 30 min, and then analyzed using Guava Easy Cyte 8 Flow Cytometer at the faculty of medicine core facility at AUB.

### MTT assay

Cell proliferation was determined by a 3-(4,5-dimethylthiazol-2-yl)-2,5-diphenyltetrazolium bromide (MTT) assay. Cells were seeded in 96-well plates (2 × 10^3^ cells/well) for 24 h before transfection with the appropriate plasmids. After 24 and 48 h of transfection 10 μl of the yellow tetrazolium salt was added to each well and incubated for 4 h at 37°C followed by the addition of 100 μl solubilizing reagent overnight. The absorbance of the developed color was measured using Multiskan EX ELISA reader at 595 nm. Cell viability was expressed as percentage viability compared to cells transfected with Lipofectamine 2000 alone, or with the corresponding empty vector. Three independent experiments were done with each condition in duplicate.

### Quantitative real-time PCR

A total of 2 μg of RNA was reverse-transcribed using iScript cDNA Synthesis Kit (Biorad) as per the manufacturer's specifications. All cDNA samples were diluted in 1:3 ratio with nuclease-free sterile water & stored at −20°C. SYBR Green Supermix (SIGMA) was used to perform the Real-Time PCR quantification by employing specific primer pairs (Supplementary Table 1) for each gene indicated while using those of GAPDH and β–ACTIN as housekeeping genes on an icycler machine (Biorad). Relative quantification was calculated using the 2^−Δ*ΔCT*^ relative quantification method.

## Results

### Expression of TBX2 family members in lung tissue and cell lines

**Figure 1:**
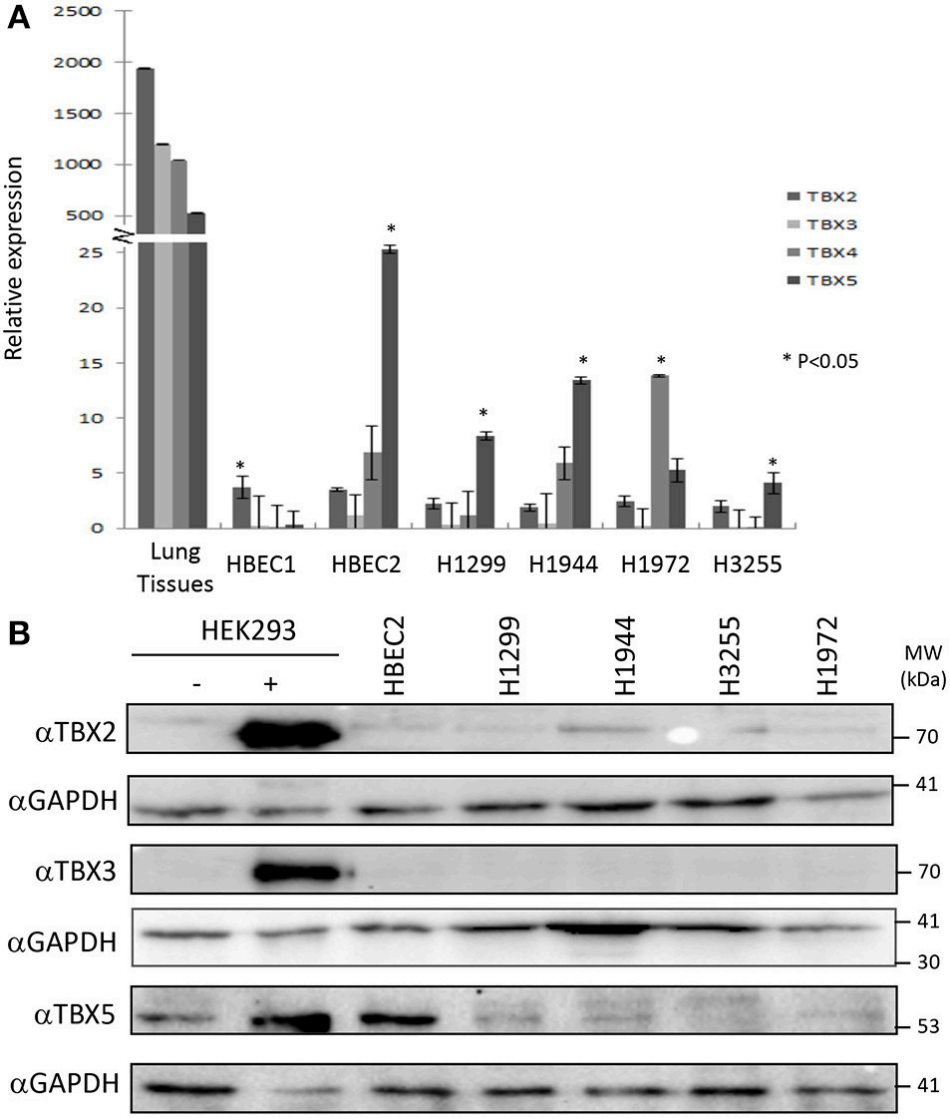
mRNA/protein profiling of TBX2 subfamily members in normal lung tissues and lung cancer cell lines. The expression of TBX2, TBX3, TBX4, and TBX5 was analyzed by real-time PCR in the lung cancer cell lines NCI-H1299, H1944, H3255, and H1972 as well as the immortalized HBEC1/HBEC2 cells and normal lung tissues. A high expression of the four members was detected in lung tissues **(A)** as compared to the different cell lines while HBEC2 cells scored the highest expression for all the four genes among the cell lines. Gene expression was first normalized to the expression of the housekeeping gene GAPDH. The fold induction of each tested gene was then normalized to the expression in NHBE cells. The data represent the means ±s.d, (*n* = 3). Significance was attributed as compared to the expression in lung tissues (*p < 0.05). The protein expression was assessed by western blotting of equal amounts of nuclear extracts from different lung cancer lines using specific antibodies for each protein **(B)**. Nuclear extracts from HEK293 cells overexpressing (+) or not (–) these proteins were used as controls.

The distribution of TBX2 and TBX5 was examined in formalin-fixed paraffin-embedded tissue sections from tumor-free regions of the lung. The sections of the lung contain branching bronchioles as well as respiratory bronchioles with adjacent alveolar walls and small to medium pulmonary arteries and veins. TBX2 showed more intense staining with prominent nuclear localization in bronchial epithelium and alveolar cells compared to that of TBX5 (Supplementary Figure 1A). In addition, TBX2 was highly expressed in the epithelial cells of the bronchioles whereas detection of expression of both TBX3 and TBX4 was not successful despite using two different antibodies (Supplementary Figure 1B). To assess the expression profile of the four TBXs in lung cancer cell lines, we designed specific primers for each member and the relative expression was measured by qPCR. The results showed the highest expression for *TBX2* in normal lung tissues followed by *TBX3, TBX4*, and then *TBX5* (Figure 1A). Immortalized human bronchial epithelial cells (HBEC2) showed a tremendous reduction in the expression of the four members as compared to that of normal lung tissues (ranging from 500 to 2,000-fold). This expression was nonetheless highly significant when compared to that of the different lung cancer lines (NCI-H1299, H1944, H1972, and H3255). For further confirmation and owing to the pronounced alternative splicing property of these TBXs, we examined their expression profile at the protein level in the different cancer cell lines as compared to nuclear extracts from transiently transfected HEK293 cells with the relative encoding plasmid. All antibodies showed high specificity while two different TBX4 antibodies didn't work at the level of western blot (Supplementary Figures 2A,B). Western blots using up to 25 μg of nuclear extracts showed very little or undetected expression of TBX2, TBX3, and TBX5 in all tested lung cancer cell lines while only HBEC2 cells expressed TBX5 which is consistent with qPCR results (Figure 1B).

### Members of TBX2 subfamily negatively affect proliferation and survival of the human NCI-H1299 and mouse MDA-F471 cell lines *in vitro*

**Figure 2:**
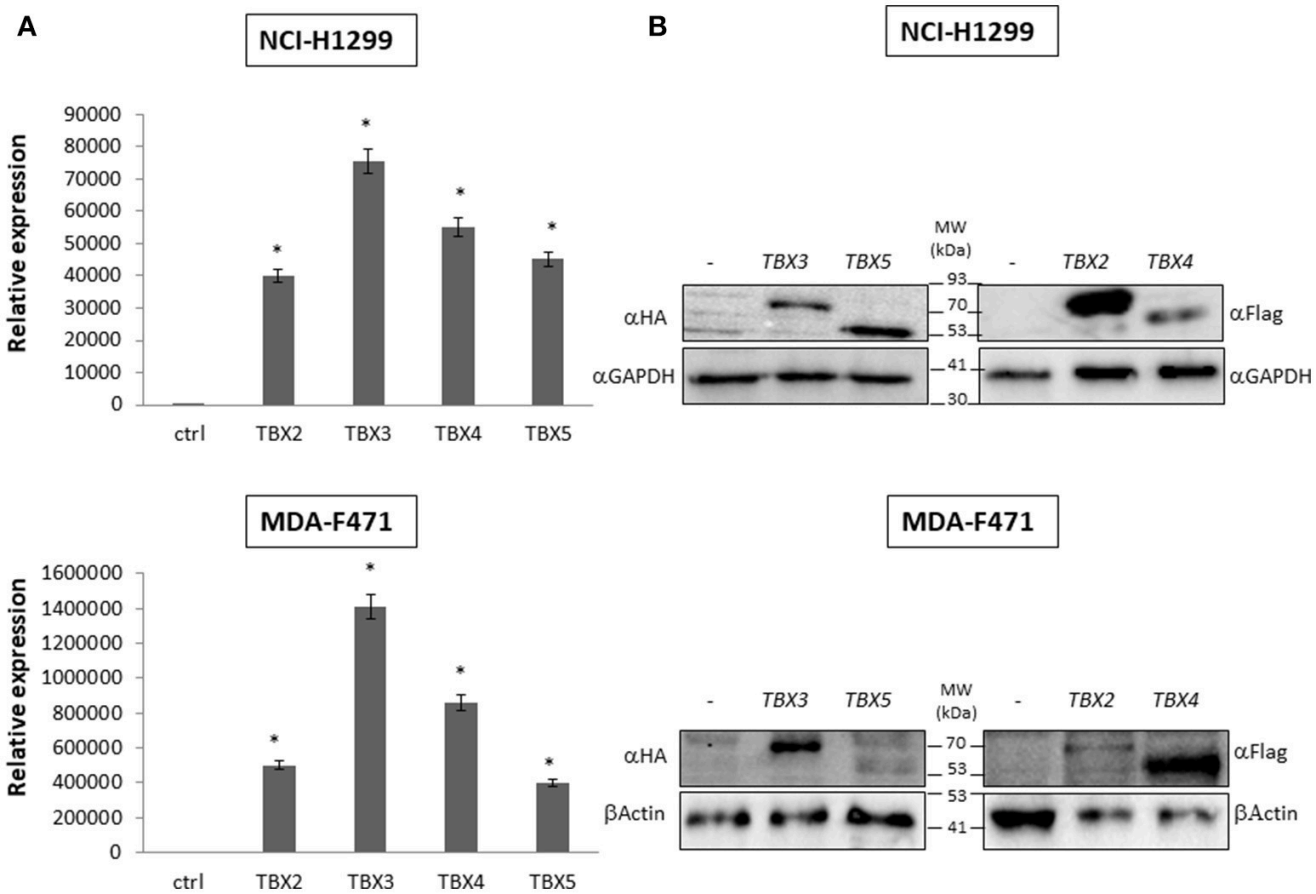
Molecular analysis of NCI-H1299 and MDA-F471 cells transiently overexpressing the different TBX proteins. Western blotting analyses and qPCR of TBX2-5 protein and mRNA expression were performed 24 h after transfection with the empty vector (–), or plasmids encoding the four members of TBX2 subfamily **(A)** For qPCR, TBXs' expression was normalized to that of the GAPDH housekeeping gene and quantified relative to the expression in cells transfected with empty vector. The data represent the means ± s.d., *n* = 2. Significance (*p < 0.05) was assessed by the Student *t*-test **(B)** For Western blotting, 20 μg of total protein from samples were analyzed by SDS-PAGE. Membranes were stained with the antibody against the corresponding tag of each vector and with a *GAPDH* or β-Actin antibody to ensure equal protein loading.

**Figure 3:**
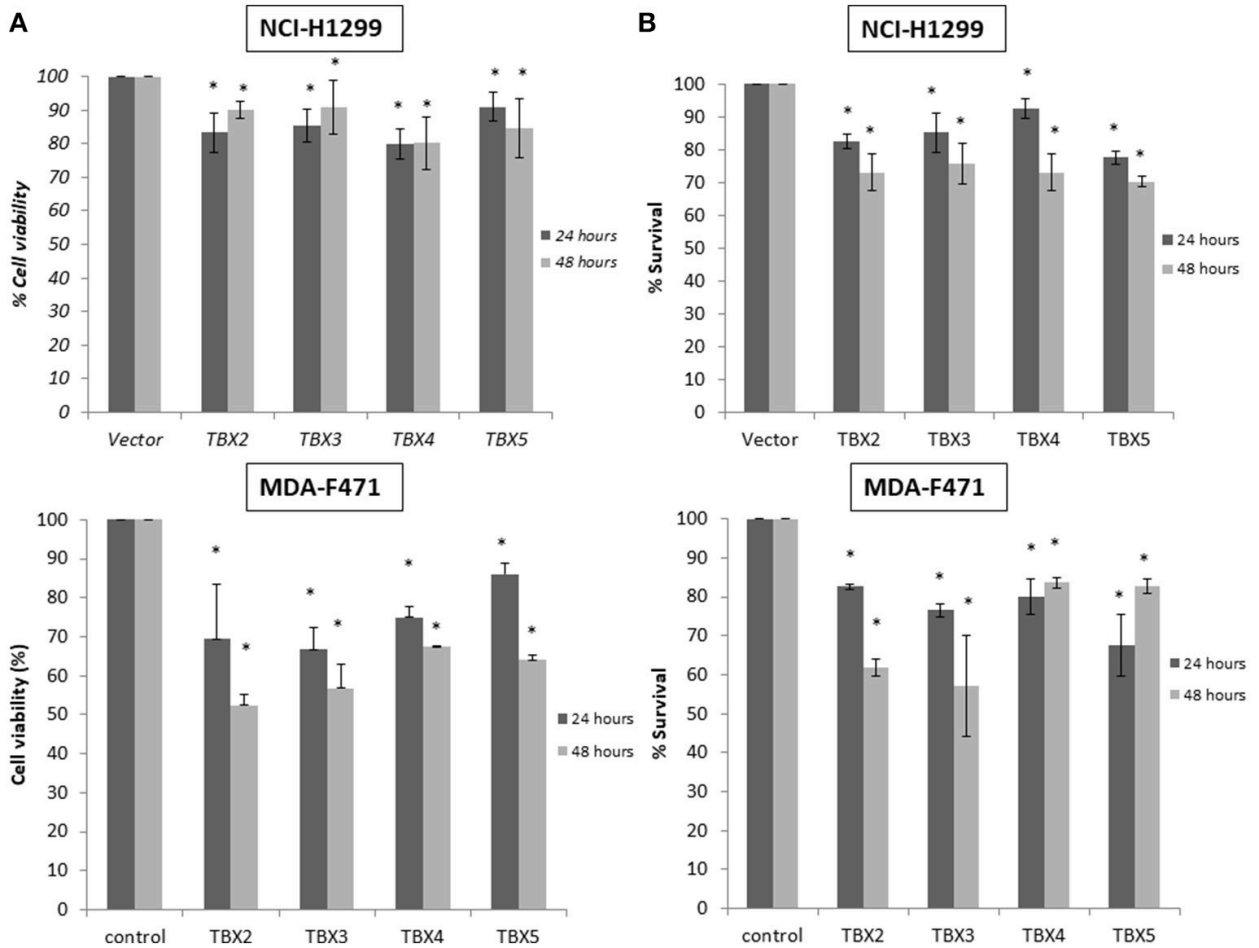
Cell viability and proliferation assays for cells overexpressing TBX2 members. Trypan blue exclusion analysis **(A)** and MTT assay **(B)** show a similar proportional reduction in cell viability with TBX2-5 transfection as compared to control in both NCI-H1299 and MDA-F471 cells. Experiments were performed at least 3 times and results are presented as the mean ± SD and are statistically (*) significant for *p* < 0.01.

**Figure 4:**
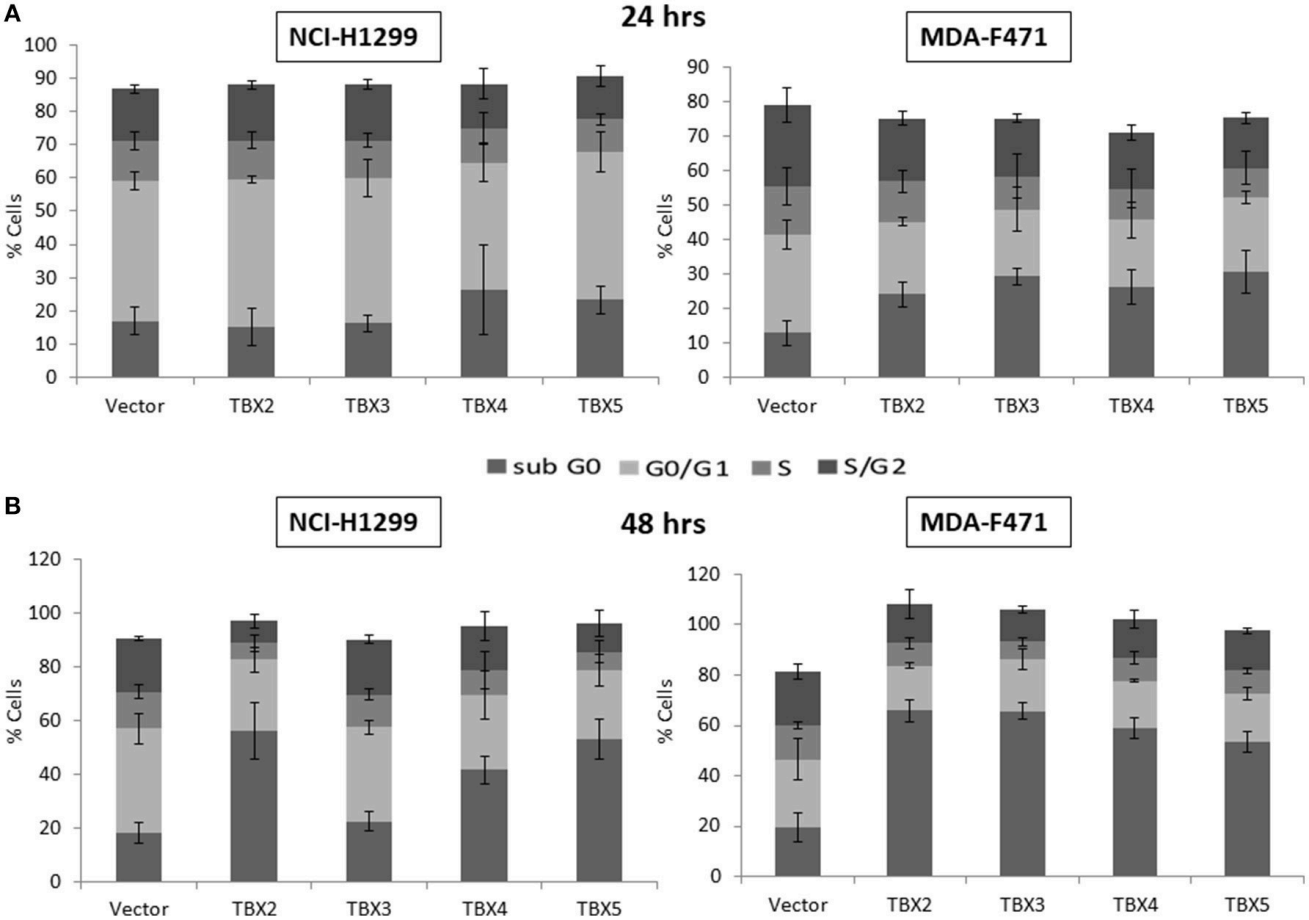
Cell cycle analysis of NCI-H1299 and MDA-F471 cells overexpressing TBX's. NCI-H1299 and MDA-F471 cells were transiently transfected with each TBX2 subfamily member encoding plasmid independently. After subsequent culture for 24 **(A)** and 48 h **(B)**, cells were stained with propidium iodide PI and analyzed for DNA content by flow cytometry. The cell cycle arrests at sub-G0 phase as a result of this transfection. Results are representative of three separate experiments. Percentage of cells in each phase (subG0, G0/G1, S, or G2/M) are presented. Data represent the mean ± SD.

We previously identified a prognostic multigene expression signature for TBX2-5 at early and advanced-stages in LUAD patients and hypothesized that these genes may function in the development and/or progression of LUADs. We thus sought to test the role of each member independently in NSCLC pathogenesis by transiently transfecting the widely used NCI-H1299 lung cancer cells and the newly derived mouse MDA-F471 mouse cells with plasmids encoding independently each *TBX* gene. Microscopic analysis of the cells showed significant cell death and decrease in proliferation as compared to the control (Supplementary Figure 3A). All cells maintained a similar morphology after transfection except those with TBX3 as they became flattened with expansion of the cytoplasm which might be a characteristic feature of cell senescence and/or differentiation (Supplementary Figure 3B). QPCR and Western blot analyses showed a significant increase in the corresponding transcript(s) and protein(s) as compared to cells transfected with the control empty vector (Figure 2). All members significantly decreased cell viability (up to 22% for NCI-H1299 and 50% for MDA-F471) as evidenced by trypan blue exclusion assay and suppressed cellular proliferation (up to 35% for NCI-H1299 and 40% for MDA-F471) as measured by MTT assay (Figures 3A,B). The pattern was quasi-identical to all members over a time course of 48 h post-transfection. To further characterize the phenotypic consequences of the overexpression, we determined its effects on cell-cycle progression by flow-cytometric evaluation of Propidium Iodide (PI) staining. Cell cycle analysis demonstrated that *TBX2-5* cause an accumulation of cells in the sub G0/G1 phase (Figure 4A), thus inhibiting their progression toward the G1 phase. This pattern was quite similar between *TBX2, TBX4*, and *TBX5* after 48 h as compared to controls ranging from 42 to 57% in NCI-H1299 and up to 60% in MDA-F471 cells. This accumulation was however less obvious with *TBX3* overexpression in NCI-H1299 (23% after 48 h) but was similar to the other TBXs in MDA-F471 cells (Figure 4B). These results suggested that *TBX2-5* induced disruption of cell cycle progression whereby the increase in sub G0/G1phase is a reflection of apoptosis induction. All together, these results prompted us to seek a molecular signature that would account for these tangible effects. We carried on RNAseq on the extracted RNAs from the above experiments each in triplicates, and we proceeded in analyzing the results taken into consideration the potential overlap in gene function as assessed morphologically and molecularly (Supplementary Figure 4).

### Genes associated with TBX'S overexpression

Pre-filtering genes with low counts across all settings (Controls; *TBX2, 3, 4, 5*) yielded 13,716 unique genes from the RNA-seq platform, which were kept for subsequent analysis. To investigate differentially expressed genes (DEGs) from the genome-wide transcriptional profiling, we analyzed each of the TBXs transfected cells separately compared to NCI-H1299 controls. The results showed a significant number of unique and overlapping DEGs, at a false-discovery rate (FDR_ threshold of 10%, across all settings (Supplementary Figures 5A,B). Interestingly, our analysis pinpointed a common molecular signature triggered by all four T-box members. This ensemble of DEGs is constituted of 112 up-regulated and 163 down-regulated genes (Supplementary Figure 6). We further compared-contrasted these common genes to the Cancer Gene Census (CGC) database that handles annotations for known oncogenes and tumor suppressors (38). Out of the 275 common DEGs, only 14 overlapped with CGC (5%; 3 up and 11 down). 9/11 down-regulated genes were annotated as oncogenes/fusion (*MTOR, LASP1, AKT2, NPM1, TRIM27, RAC1, MLLT3, EIF1AX, NONO*) and 2/11 as tumor suppressors (*NF2* and *ERCC2*). All three up-regulated genes (*CHD2, TET2, TNFAIP3*) were annotated as tumor suppressors. The full list of common up/down regulated genes, and corresponding annotation from CGC, is provided in Supplementary Table 2.

### Methylation/demethylation pathways are directly affected by TBXs overexpression in NCI-H1299 cells

**Figure 5:**
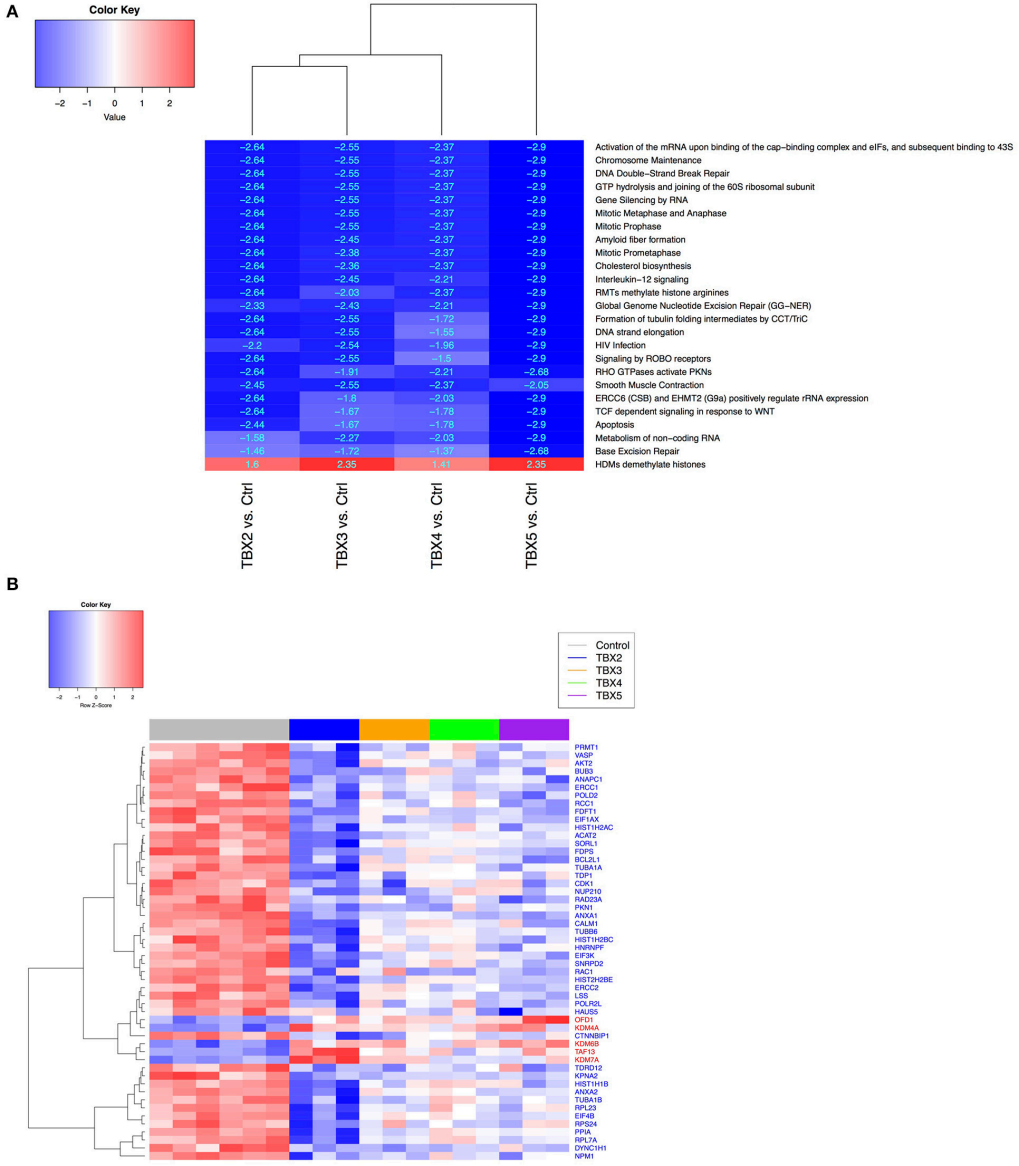
Comparison of Reactome pathways **(A)** and gene expression data **(B)** between control H1299 and TBX construct groups. **(A)** Shared and significant Reactome pathways across all contrasts (TBX2-5 vs. controls, FDR < 0.05), from the gene set enrichment analysis (GSEA). Pathway labels: red (*N* = 1 up-regulated), blue (*N* = 24 down-regulated). For each pathway, numerical values represent the signed enrichment score (ES) multiplied by the negative log10 of the corresponding *p*-value **(B)** Heatmap showing the leading-edge genes (*N* = 52), from the rank-based pathway enrichment analysis approach, extracted from **(A)** and differentially expressed in all TBX contrasts (FDR < 0.1 from DESeq analysis). Gene labels: red (*N* = 5 up-regulated), blue (*N* = 47 down-regulated).

**Figure 6:**
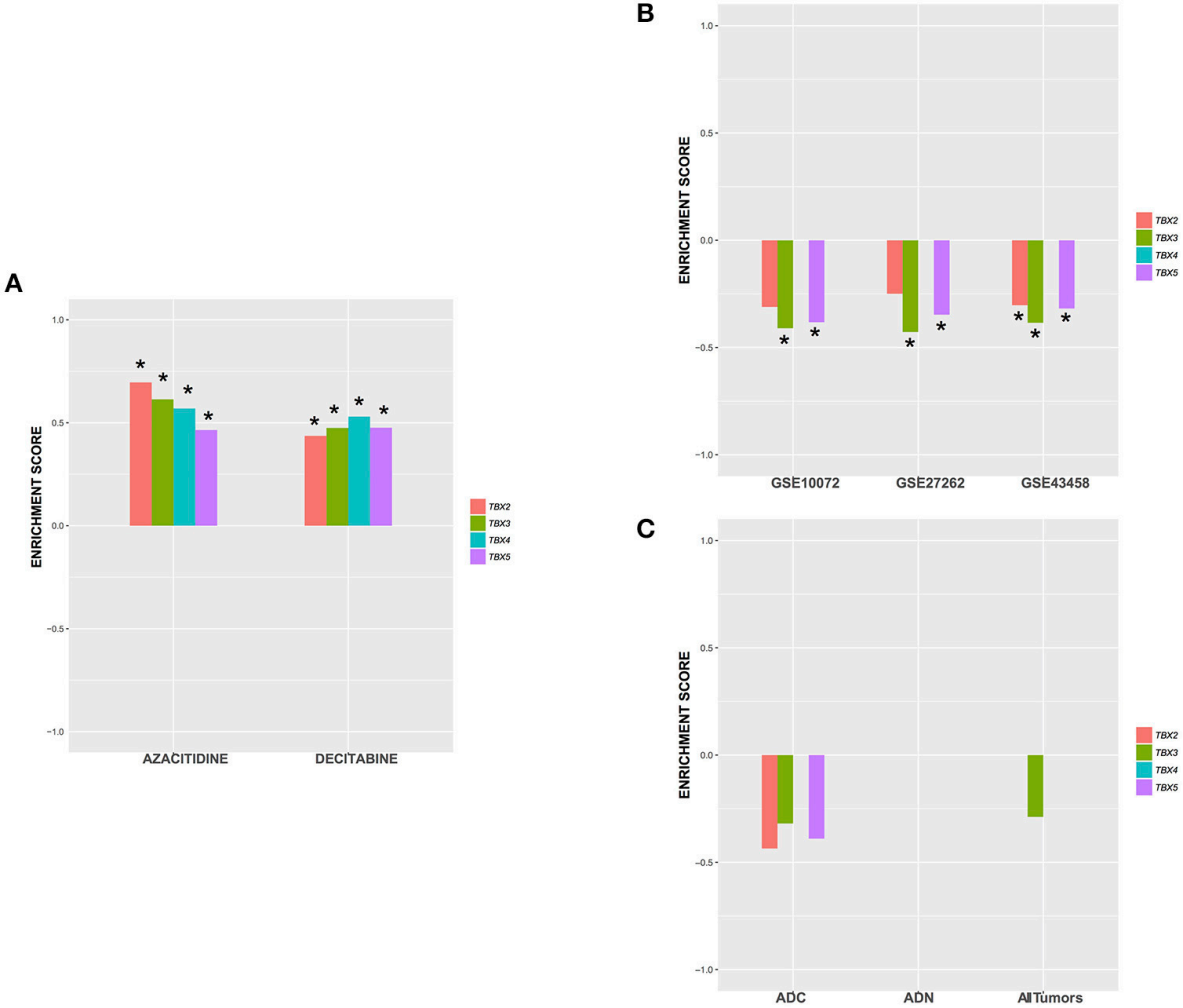
Enrichment score of the top 75up /75 down-regulated genes in all TBX settings against signatures from **(A)** NCI-H1299 cells treated with 2 demethylating agents (GSE29077), **(B)** 3 lung adenocarcinoma public datasets, and **(C)** mouse adenocarcinomas from *Gprc5a* knock-out mice. The plots show the enrichment score for each setting ∈ [−1,1]. A positive enrichment score suggests a similar mechanism of action while a negative one would suggest an opposite/dissimilar molecular perturbation. *Significance level (*p* < 0.05). ADC, adenocarcinoma; AND, adenoma.

Based on the ontology of the affected genes, we questioned their interconnectivity in global cellular pathways. We thus leveraged an unbiased bottom-up approach to unveil molecular perturbations induced by the TBX2 family members genes. We ranked all the genes according to the t-stat from each of the differential expression analysis with respect to the two considered phenotypes (TBXs vs. control). We then run a pre-ranked gene set enrichment analysis using Reactome pathways (39, 40). After filtering all highly redundant pathways, we only kept the top 25 with and FDR < 0.1 showing consistent enrichment scores across all four settings (*TBX2* vs. control, *TBX3* vs. control, *TBX4* vs. control, and *TBX5* vs. control). The results showed that most of the common pathways are down-regulated and are linked to cell cycle and DNA replication mechanisms confirming thus our microscopic and molecular data (Figure 5A). Interestingly, only one unique pathway “HDMs demethylate histones,” was up-regulated by all TBXs. Furthermore, we questioned the overlap between the genes from the 25 significant pathways and the list of commonly regulated genes obtained previously (Figure 5A and Supplementary Table 3). We found that ~19% of those genes (52/275) were in common, and notably those involved in the histone demethylation pathway, like *KDM4A, KDM6B*, and *KDM7A* which are known to reverse N-methylations of histones (Figure 5B). To further establish that demethylation is an important molecular hallmark induced by the TBX2 subfamily in NCI-H1299 cells, we used a pattern matching algorithm, similar to a previously described approach in order to compare our molecular signature to an expression profiling panel from two known DNA methyltransferase inhibitors, azacitidine and decitabine (41). Our results showed that both signatures share a highly correlated set of genes (*p* < 0.01) (Figure 6A and Supplementary Table 4). This observation suggests that the anti-proliferative potential of T-boxes in NCI-H1299 is mainly a consequence of epigenetic remodeling. Validation for expression changes in selected transcripts was done using qPCR specific primers for genes involved in regulating cell cycle progression (*MTOR*), methylation (*DNMT1*, and *KDM6B*), proliferation (*AXL*), and apoptosis (*EGR1, FOS*). Data analysis indicated that the results from qPCR were consistent with the RNA sequencing data (Supplementary Figure 7).

### The *in vitro* TBX overexpressed molecular signature is highly anti-correlated to that of the *in vivo* lung adenocarcinoma

Based on our previous findings, we hypothesized that overexpression of TBX2 family members in lung cancer would restore a “normal” pulmonary cellular milieu. We thus compared our ranked list of differentially expressed genes *in vitro* to gene expression profiling of lung adenocarcinomas and normal lung tissue from three clinical cohorts GSE43458, GSE27262, GSE10072 using the same pattern matching algorithm as described previously (Figure 6B). Our results showed that signatures from *TBX3* and *TBX5* are significantly anti-correlated with lung adenocarcinoma signatures from patients (*p* < 0.05). Interestingly, the *TBX2* signature is the least significant while that of *TBX4* exhibits no changes across all settings (Figure 6B, and Supplementary Table 5). The inhibition of growth, proliferation and cell cycle progression might be considered as the molecular events associated with the cytotoxic activities and epigenetic modifications' effect of the four members of the *TBX2* subfamily in the mouse cells as in the human cells. To test this hypothesis, we used the same anti-correlation bioinformatics approach on the RNA datasets generated from the *Gprc5a* nicotine-induced lung cancer model. Interestingly, only gene signatures from mouse adenocarcinomas were anti-correlated with TBX's induced signatures (Figure 6C). Enrichment scores were negative but not significant with *TBX4* falling apart, and *TBX2* scoring the best followed by *TBX3* and *TBX5* (Figure 6C and Supplementary Table 6).

## Discussion

The T-box family of transcription factors exhibits an evolutionary conserved pattern across mammalian species. In humans, several mutations in the T-box DNA binding domain is associated with developmental defects, and recent evidence from our lab showed that *TBX2 s*ubfamily (*TBXs 2, 3, 4*, and *5*) was significantly repressed in NSCLC, which warranted further investigation in lung cancer (26). Curing the most fatal cancer in the world and finding novel, personalized, and/or combined therapies is still the most challenging goal in this area. This is why we interrogated the potential use of TBXs as tumor suppressor proteins in order to unravel novel mechanisms that could be therapeutically targeted. We investigated the extent to which overexpressing these transcription factors would abrogate cell proliferation in NCI-H1299 lung adenocarcinoma cell line. Our bioinformatics' analyses concurred with phenotypic screening and showed a significant decrease in cell cycle pathways. Interestingly, molecular signatures from TBXs resembled gene signatures induced by hypomethylating agents, which further support the potential use of epigenetic therapy in lung adenocarcinoma.

The first paradigm in our data was the proof that most lung cancer cell lines do not express significant amount of TBX2-5 mRNA and/or protein. Not only we confirmed the consistent suppression of expression of all 4 genes in all tested lung cancer lines, but we provided a proof of their robust expression in normal lung adult tissues. Interestingly, this expression at least for TBX2 as evidenced by immunostaining is not only restricted to mesenchymal cells, but also extends toward most of the cells including the epithelial cells of the bronchi. We thus consolidated our previous findings by providing indirect evidence that the downregulation of *TBX2-5* in LUADs is not due to the expansion of negatively expressing TBX cells but is rather due to a cell-autonomous regulated mechanism, and thus the TBX2 family signature could be a reliable testing biomarker for LUADs. While this downregulation is still to be tested in conditional knock-out models of *Tbx2-5* in order to confirm its sufficiency in developing spontaneous or nicotine-induced LUADs, the results of our transient gain of function experiments show that these transcription factors can function independently to stop the progression of lung cancer. By inhibiting cell proliferation and inducing apoptosis, we postulate that all 4 members converge independently into a common pathway that would inhibit lung cancer progression as opposed to their role in expanding the lung cell lineage pool in early pulmonary development and subsequent morphogenesis. This apparent contradiction finding is however corroborated by recent studies in different cancer cell lines, including non-small cell lung carcinoma (NSCLC) cell lines where overexpression of TBX5 was shown to suppress cell proliferation, colony formation, and invasion and to induce apoptosis (42). While no studies have previously been published in other cancer cell lines for transient overexpression of *TBX4* and *5*, numerous studies have shown that transient overexpression of *TBX2* or *TBX3* does specifically affect senescence by targeting the tumor suppressor gene *CDKN1A* and *B*, and *CDKN2A* (19, 43). Our results show a very slight activation of the latter genes but was not significant enough to rank these factors amongst the top deregulated genes, as contrasted to the major suppressive effect underscored on genes implicated in cell proliferation. One potential explanation might be linked to the cellular context, i.e., the NCI-H1299 lung cancer cell line we utilized is p53 defective. However, our unpublished results in other lung cancer cell lines, and the results obtained by overexpressing *TBX5* in A549 and NCI-H596 cells by Ma et al. do not support this hypothesis (42). Indeed, our RNAseq approach is the first documented unbiased approach to assess the role of *TBX2-5* in a cancer setting as opposed to previous studies were “a priori” and specific knowledge were utilized. It helped thus in studying the global effect of these genes while detecting a common altered top-ranked genes' set which anti-correlated with that of LUADs datasets we previously used to confirm a suppression in the 4 *TBX* genes in adenocarcinoma. Moreover, this detected set of top deregulated genes anti-correlated with our documented *Gprc5a*^−/−^ murine model of nicotine-induced lung adenocarcinoma, suggesting not only a tumor-suppression potency linked to the expression of these TBX genes but also a tumor malignancy reversal gene switch. What makes the data more challenging is the striking similarity between all TBX family members as highlighted by the pre-ranked gene set enrichment analysis we did using the Reactome pathways which showed a marked inhibition for numerous paths linked to cell proliferation, chromosome maintenance, and apoptosis.

The second paradigm of the analysis was the finding of only one commonly activated pathway that involve the histone demethylases. This activation is also congruent with the downregulation of the RMTs and thus could be the key to explain most of the downstream effects.

Activation of the main histone-demethylases *KDM4A, KDM6B*, and *KDM7A* coupled to the repression of some protein arginine methyltransferases encoding genes like *PRMT1* by all TBX2 proteins suggested that these events proceed all other related or unrelated events like cell proliferation and apoptosis. Although we do not exclude that each TBX gene has a unique signature and might exert its effect independently as per our analysis of the top-ranked genes in each setting alone (Supplementary Figure 5), we do believe that the methylation/demethylation pathway is a plausible gating channel for all other events. The reasoning underlying this statement stems from the following observations: 1- The T-box domain potency to directly interact with some histone methylases and demethylases (44); 2- The sufficiency of the T-box domain of *TBX2* to directly bind to the H3 Histone and recognize mitotic chromatin (45). While the latter observation would explain how these T-box proteins can directly bind to their targets and activate or repress transcription in a high-mitotic indexed milieu, the former does provide explanation of how global demethylation could be restored by repressing the expression and activities of PRMTs and inducing the expressing and enhancing the activities of KDMs. In that regard, *TBX21* was the first T-box family member to be dissected toward its potential interaction with epigenetic modulators (46, 47). The documented results underscore the importance of two highly conserved regions with the T-box DNA binding domain to physically interact with both the *NCOR2* methyl-transferase and *KDM6B* dimethyl transferase (48). The molar distribution of such keys proteins at a given time during development and/or in a cellular pathological condition will thus dictate the output of the interaction by means of evaluating the transcriptional activity of TBX21. Interestingly, the amino acids implicated in the docking mainly to the demethylase moiety are highly conserved between members of the T-box family of genes. A recent survey of the mutations occurring within the T-box domain in those *TBX* genes known to cause the different genetic syndromes did show that they abrogate either the interaction with the known methyl or dimethyl transferases (47). However, the direct supporting evidence to our data came from a study that showed using a newly described screening system that *TBX5* is one of few transcription factors that induce binding site-directed DNA demethylation (49). These findings coupled to the global methylation process that underscores all lung cancer subtypes and mainly the nicotine-induced types will thus potentially position the effect of the for *TBX* genes in the pathway that includes demethylating agent in clinical trials like 5-AZA (50, 51). Indeed, our top ranked genes and gene signature were significantly correlated with those derived from the NCI-H1299 cells treated with azacitidine and decitabine. While we don't exclude other pathways like those linked to the immune-therapy like *AXL* downregulation (52), to be of equal importance to the methylation/demethylation pathway, we are confident that those additional pathways would be linked to individual TBX2 family members rather than being shared as per our analysis of gene and pathway enrichments (Supplementary Figure 5).

## Conclusion

Our results are the first to shed lights on the role of TBX2 family members in lung tumor suppression and epigenetic remodeling in NSCLC. The four members of this family were able to suppress cell cycle progression through regulating the methylation /demethylation process which in turn influence the expression of the downstream genes that are implicated in cancer development and progression. Reactivation of TBX2 subfamily in LUAD could thus represent a major and specific therapeutic avenue for reversing the molecular profile in lung tumor.

## Availability of data and materials

All data are available for the scientific community, including raw genetic RNAseq data would be uploaded to the Gene Expression Omnibus (GEO).

## Author contributions

AK and BD did the experimental work and participated in the analysis of the results. NE-H did the analysis and interpretation of the RNAseq data. FB, AK, and GN interpreted the histological findings. JK, AS, JF, and HK did the initial work on the mice Gprc5α knockout. AK, NE-H, and GN wrote the paper draft, and all authors contributed to the analysis of the results, interpretation of the data, and final write up of the paper. GN, wrote, and secured funding for the project.

### Conflict of interest statement

AS is a founder and consultant of Allegro Diagnostics, Inc. and is a consultant for Veracyte, Inc. and Janssen Pharmaceuticals. The remaining authors declare that the research was conducted in the absence of any commercial or financial relationships that could be construed as a potential conflict of interest.

## Supplementary material

Supplementary Figure 1 Expression of TBX2 and 5 in adult human lung tissue. The αTBX2 stained slide shows nuclear and cytoplasmic staining (brown color) in bronchial epithelium (arrows in **B**) and alveolar lining (pneumocytes) with more prominent nuclear staining. While αTBX5 shows a staining within bronchial epithelium but it is less intense andlacks differential intensity between nucleus and cytoplasm; this is in contrast with the IgG negative control **(A)**. Magnification X5 **(A)** and X20 **(B)**.

Supplementary Figure 2 Specificity of the TBX2/3/4/5 antibodies. **(A)** Western Blots showing the overexpressed TBX2,3, and 5 proteins only recognized by the corresponding antibody (αTBX, αTBX3, αTBX5). None of the antibodies recognize a different TBX protein than the one it was raised against **(**Left panel**)**. Two different antibodies against TBX4 were used and neither one worked at the level of western blot as confirmed by using the anti-Flag antibody (TBX4 is Flag tagged) that confirmed the transfection and the overexpression process **(**right panel**). (B)** Immunofluorescent assay: The corresponding vectors were independently transfected in HEK293 cells which don't show an endogenous expression of these proteins. The four antibodies were able detect their targets as represented by the red color. Images were taken using the oil-immersed X40 magnification objective of a Carl Zeiss LSM 710 laser scanning confocal microscope.

Supplementary Figure 3 Microscopic evaluation of the NCI-H1299 cells overexpressing TBX2 family members. **(A)** By Phase contrast microscopy, cells were visualized 24 h post-transfection with TBX2/3/4/5 vectors independently as compared to cells transfected with empty vector or those only using the transfecting agent (lipofectamine 2000). Images were taken using a X10 magnification objective. **(B)** By Confocal microscopy using the Carl Zeiss Zeiss LSM 710 microscope, all TBXs overexpressing cells showed mainly a nuclear localization except for TBX3 (red color) with relative reduction in Ki-67 intensity (green) in transfected cells compared to non-transfected cells. Nuclei are stained with Hoechst (blue). Images were taken with a X40 oil objective.

Supplementary Figure 4 Analysis workflow for the NCI-H1299 transfected cells with TBX gene expression constructs. A comparative analysis of the four experiments (TBX2,3,4,5) was conducted by investigating differentially-induced pathways in each experiment vs. control NCI-H1299 lung cancer cells. Genes were ranked to perform a gene set enrichment analysis (GSEA) on Reactome pathways. The same GSEA pattern matching technique was performed to assess connectivity of the top 75 up/75 down-regulated gene signature from each of the experiment against differentially expressed gene sets from GEO lung adenocarcinoma datasets (see Material and Methods section).

Supplementary Figure 5 Unique and overlapping deregulated genes in NCI-H1299 cells overexpressing TBX2-5. Venn diagrams showing the overlap of significant down-regulated **(A)** and upregulated genes **(B)** in all experimental contrasts (FDR < 0.1).

Supplementary Figure 6 Heatmap of gene expression comparisons between control cell lines and TBX construct groups. The top 275 significantly regulated genes common to all TBXs contrasts (FDR < 0.1) are shown with the red labels for up-regulated (112 genes), and blue labels for down-regulated (163 genes).

Supplementary Figure 7 Confirmation of RNAseq data by qPCR analysis. *ACTN1*,*AXL*,*DNMT1, EGR1*, FOS, EZH1,*KDM6B* and *MTOR* genes in H1299 lung cancer cells transfected with control and TBX2/3/4/5 plasmids. Expression changes are depicted relative to cells transfected with control plasmid. ^*^significance (*p* < 0.05 assessed by the Student's *t*-test).

Supplementary Table 1 List of genes with their corresponding primers used in qPCR.

Supplementary Table 2 Common deregulated genes with their expression change and CGC annotation.

Supplementary Table 3 Common Regulated Genes in Reactome Pathways Deregulated in NCI-H1299 cells overexpressing TBXs.

Supplementary Table 4 Enrichment scores and corresponding p-value from the 75 most up-regulated and down-regulated genes in TBXs with respect to ranked gene signatures from hypomethylating agents.

Supplementary Table 5 Enrichment scores and corresponding p-value from the 75 most up-regulated and down-regulated genes in TBXs with respect to ranked gene signatures from public data in GEO.

Supplementary Table 6 Enrichment scores and corresponding p-value from the 75 most up-regulated and down-regulated genes in TBXs with respect to ranked gene signatures from a murine tumor model dataset.
