# Supplementary material for: Transcriptomic Alterations in Lung Adenocarcinoma Unveil New Mechanisms Targeted by the TBX2 Subfamily of Tumor Suppressor Genes
Source: Front Oncol. 2018 Oct 30;8:482. doi: 10.3389/fonc.2018.00482 (PMC6218583; doi:10.3389/fonc.2018.00482)

Supplementary Figure 1

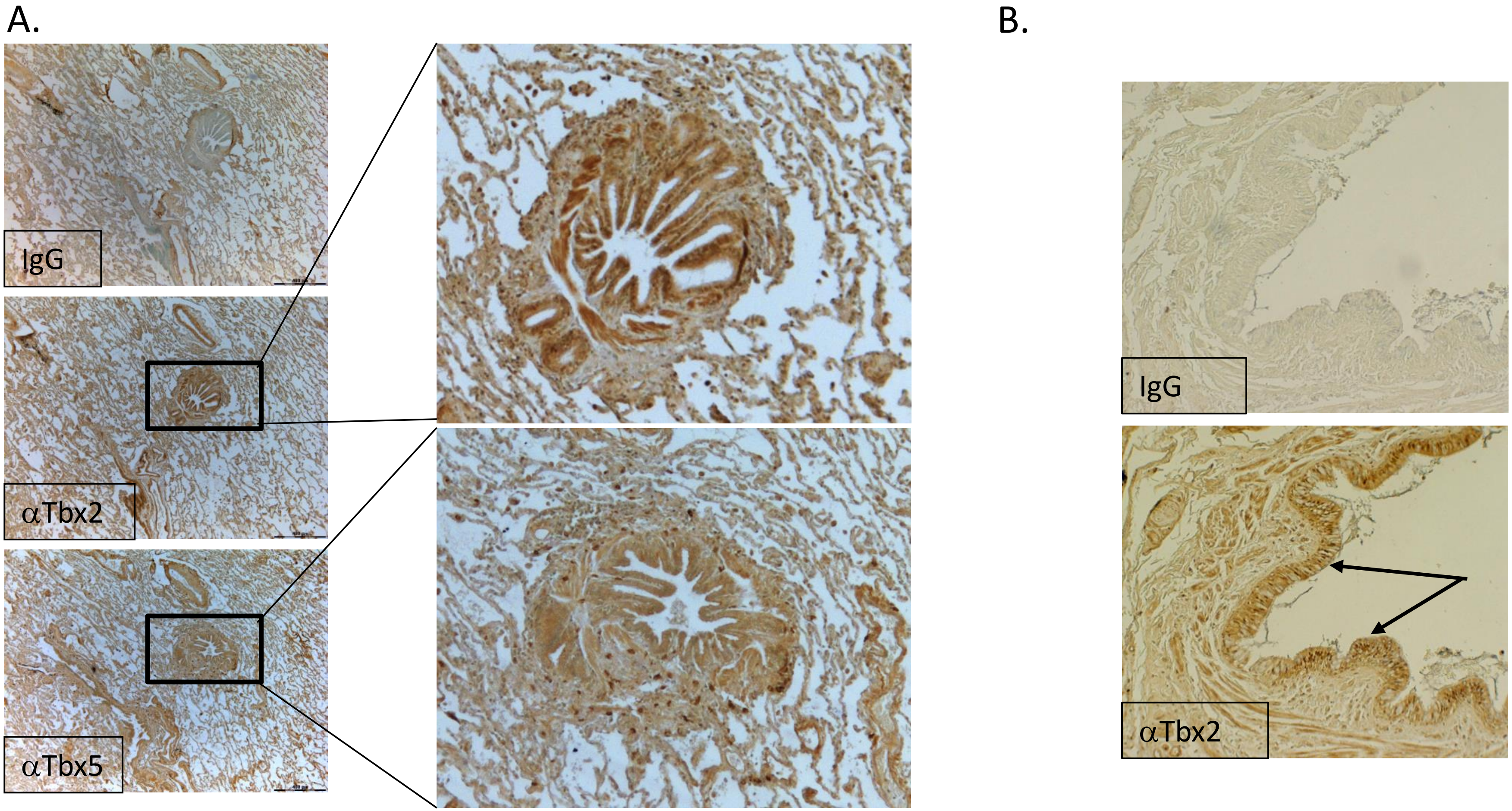

Supplementary Figure 2A

A.

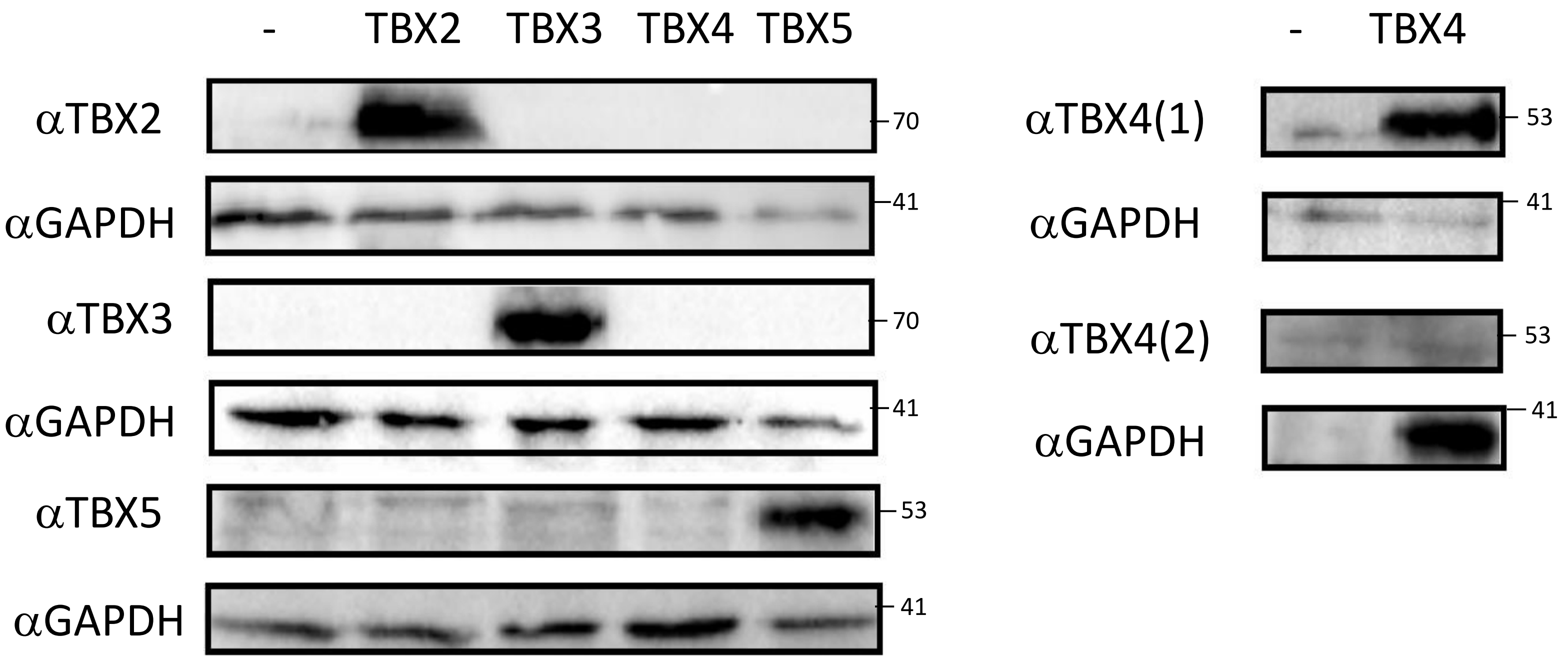

Supplementary Figure 2B

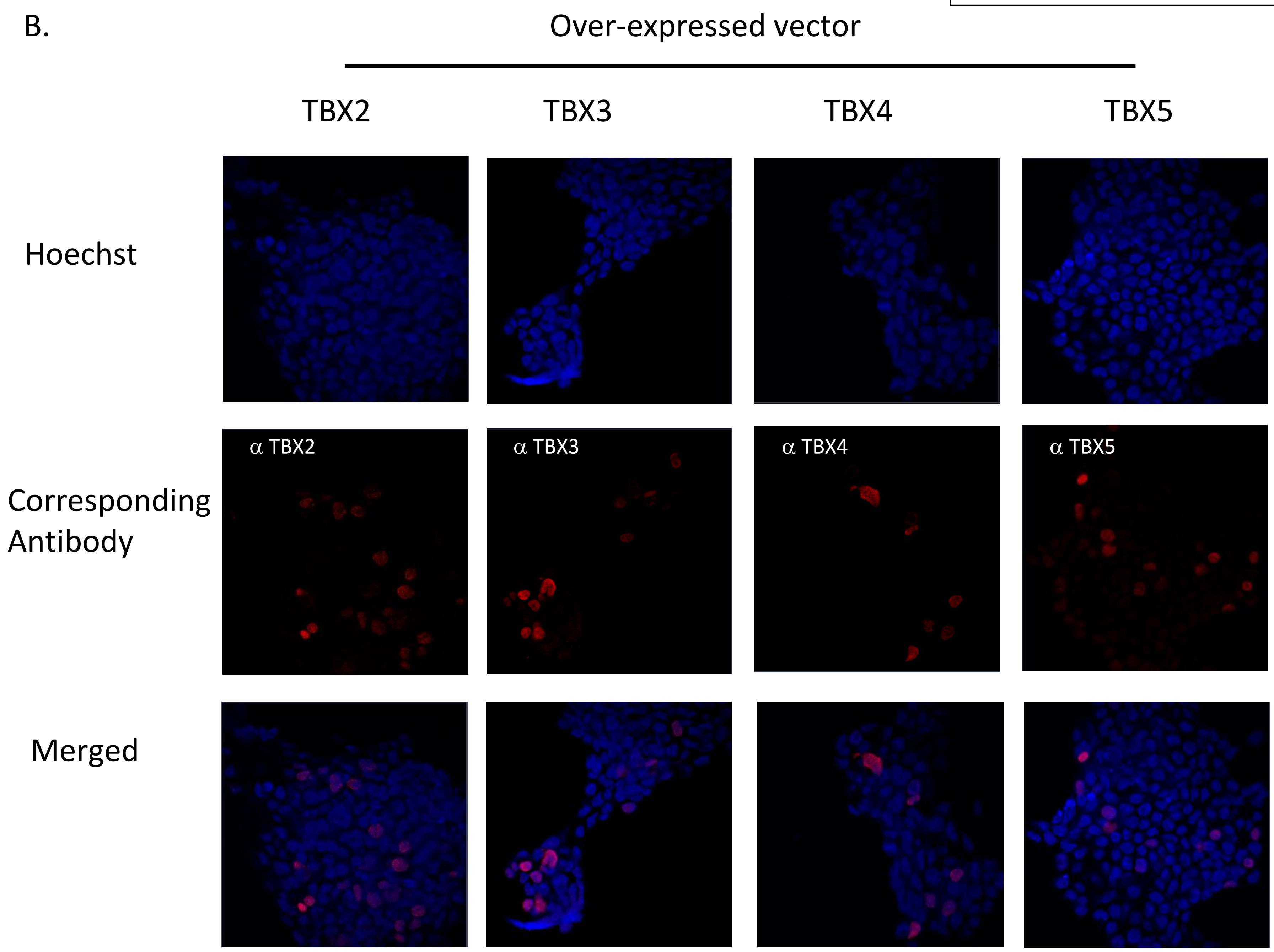

Supplementary Figure 3A

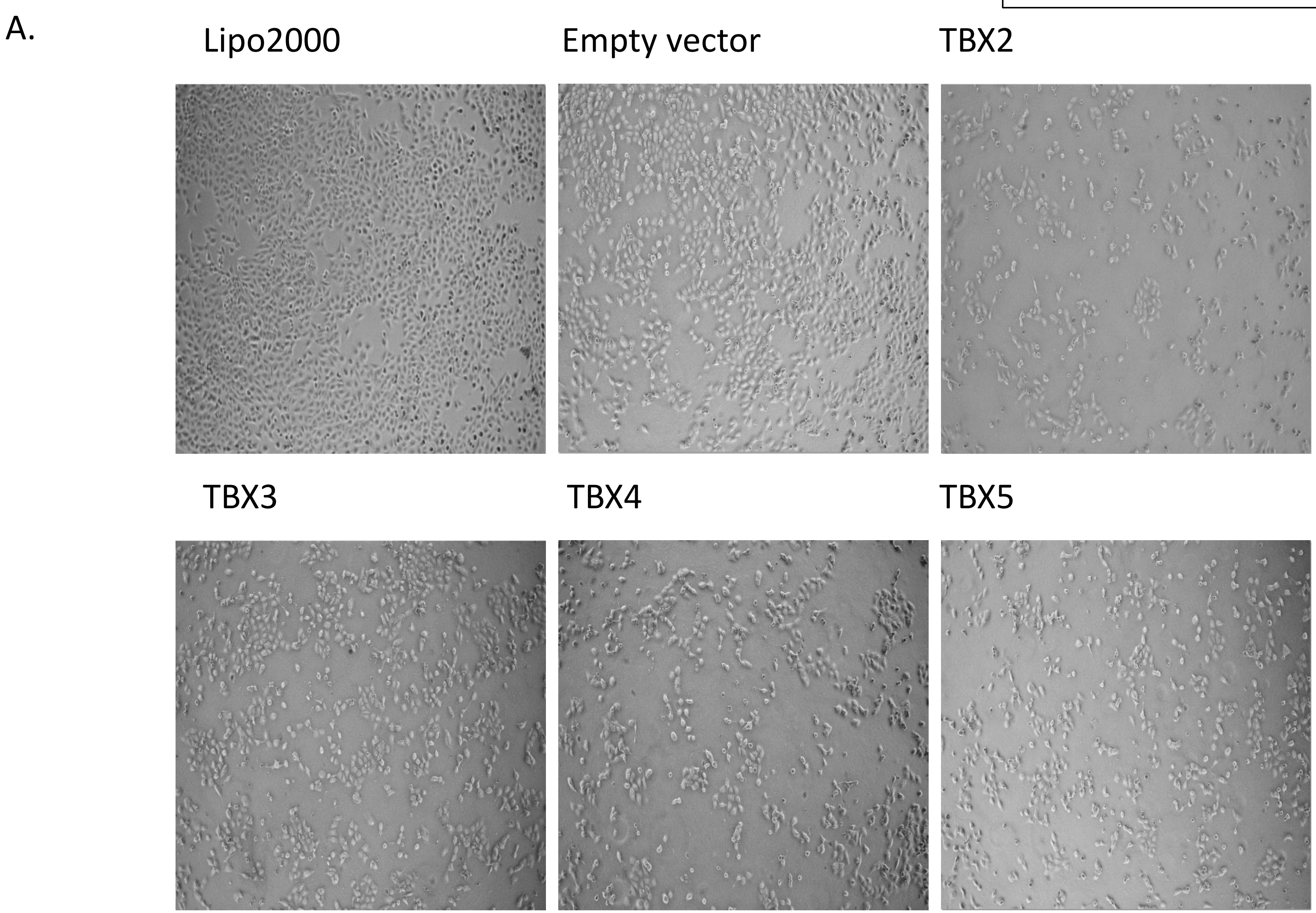

Supplementary Figure 3B

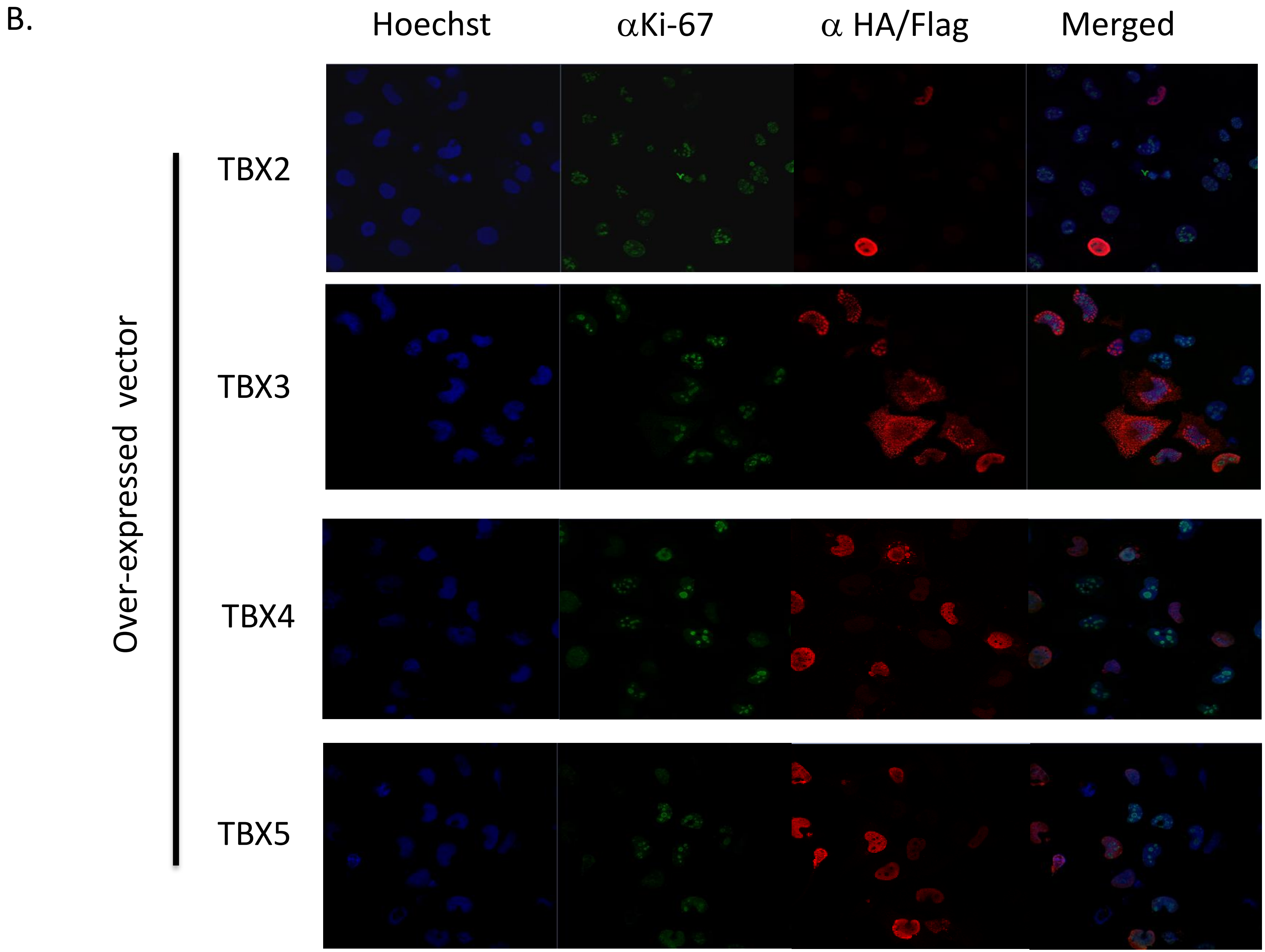

Supplementary Figure 4

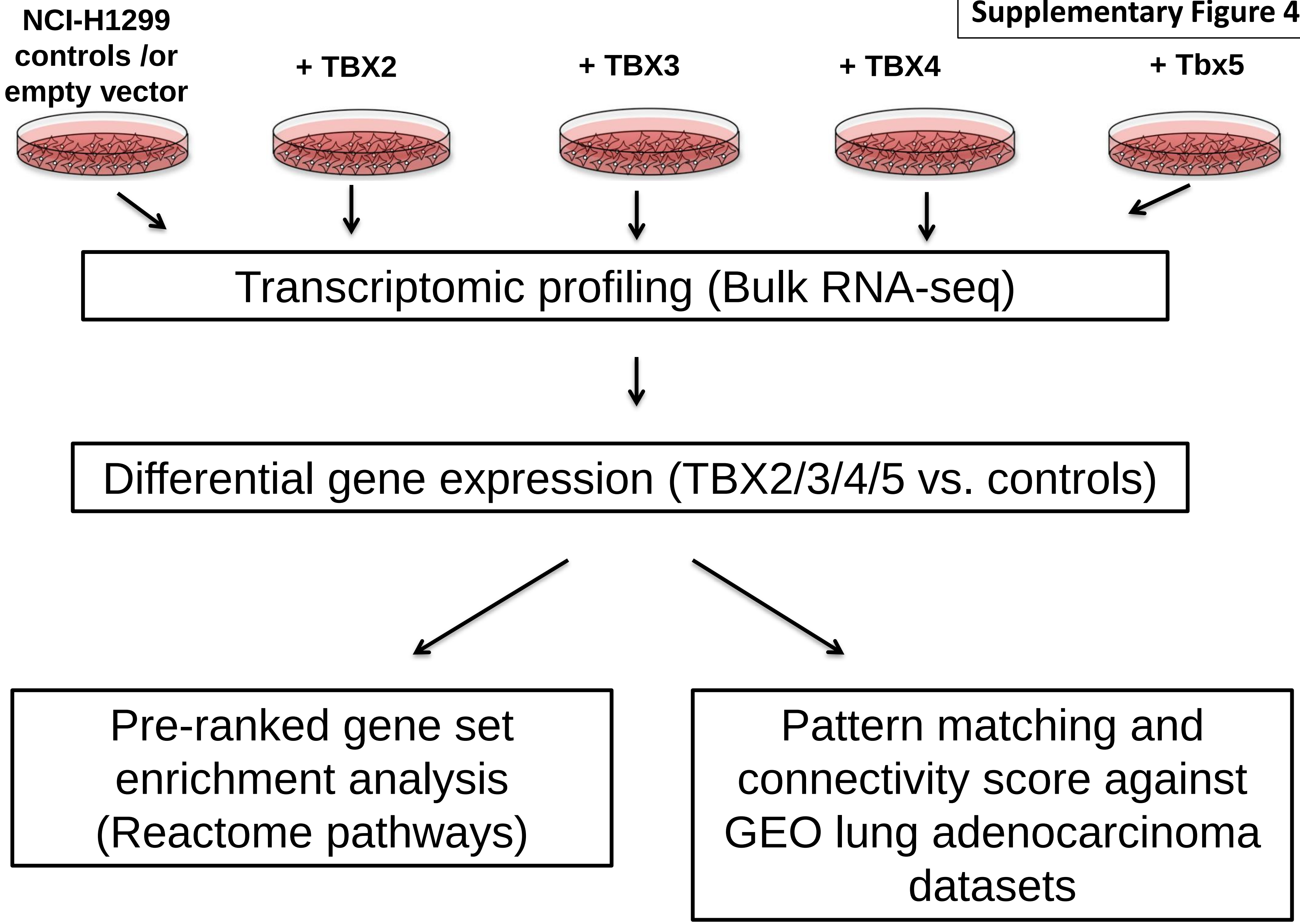

Supplementary Figure 5

A.

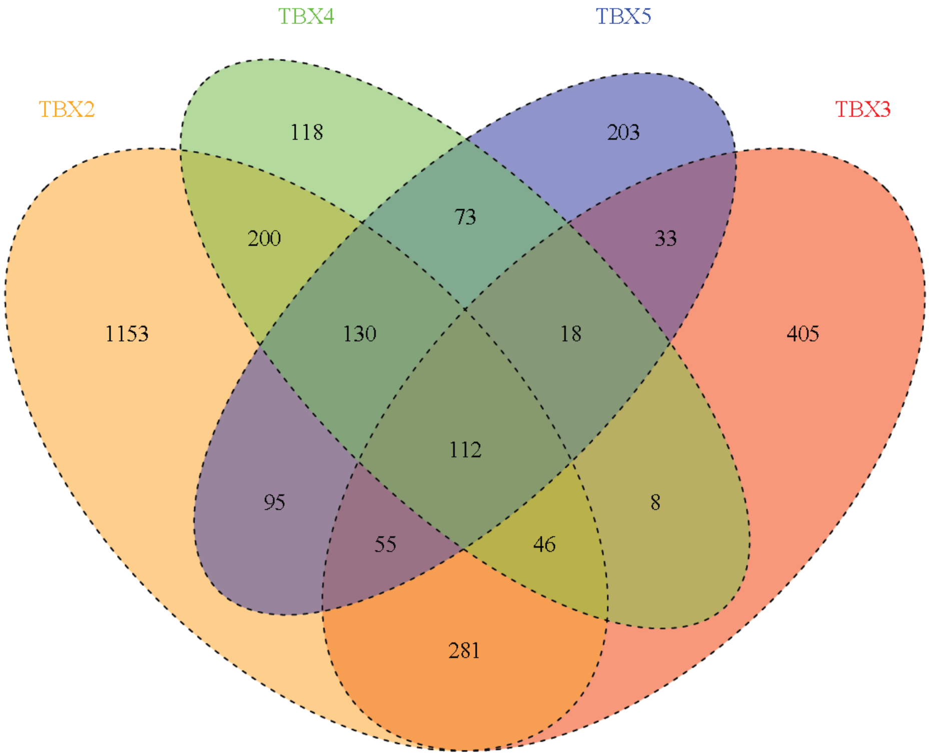

B.

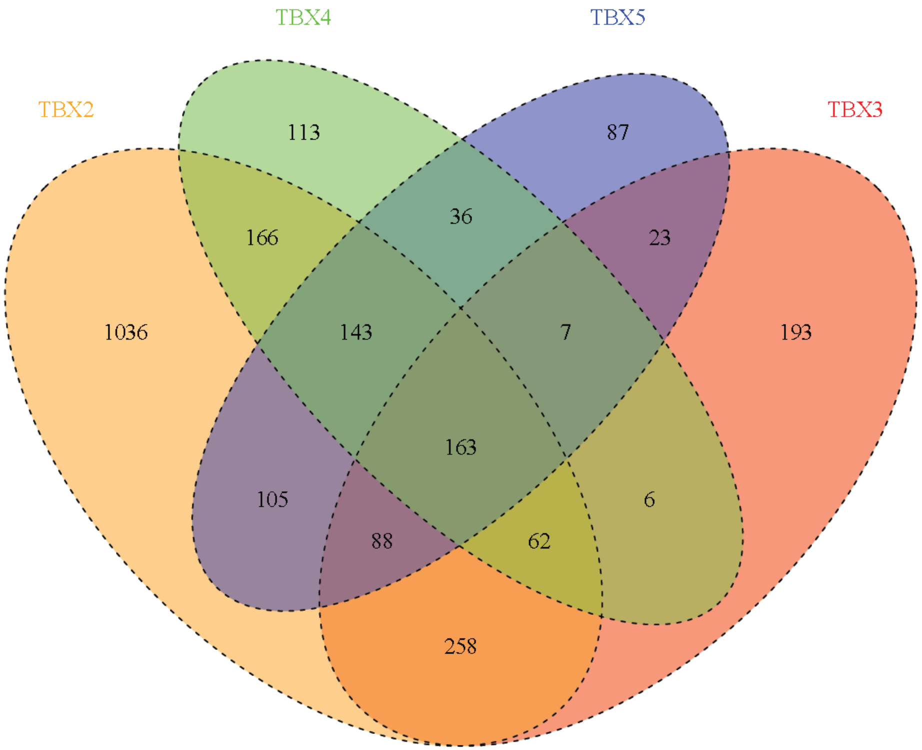

Supplementary Figure 6

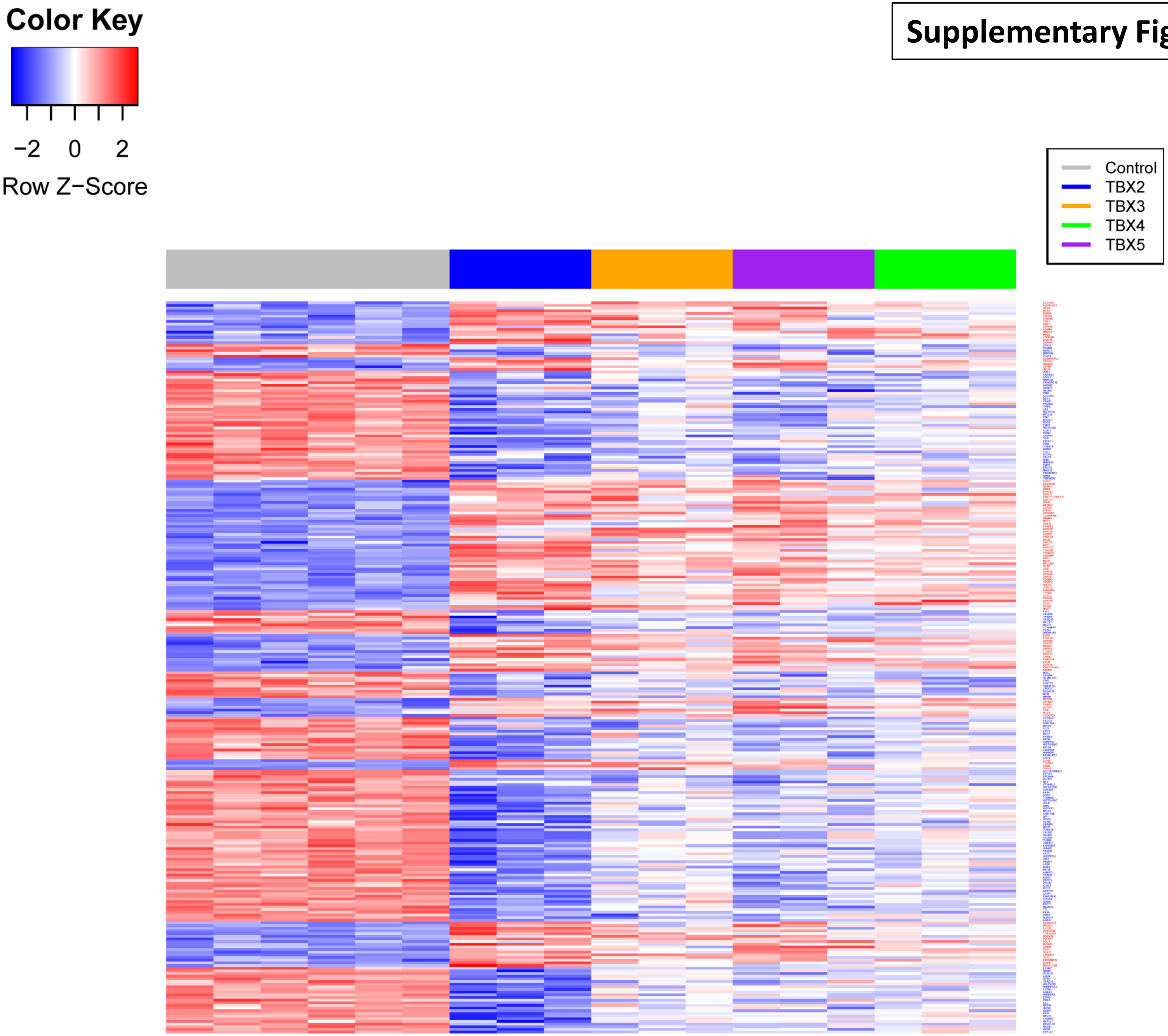

Supplementary Figure 7

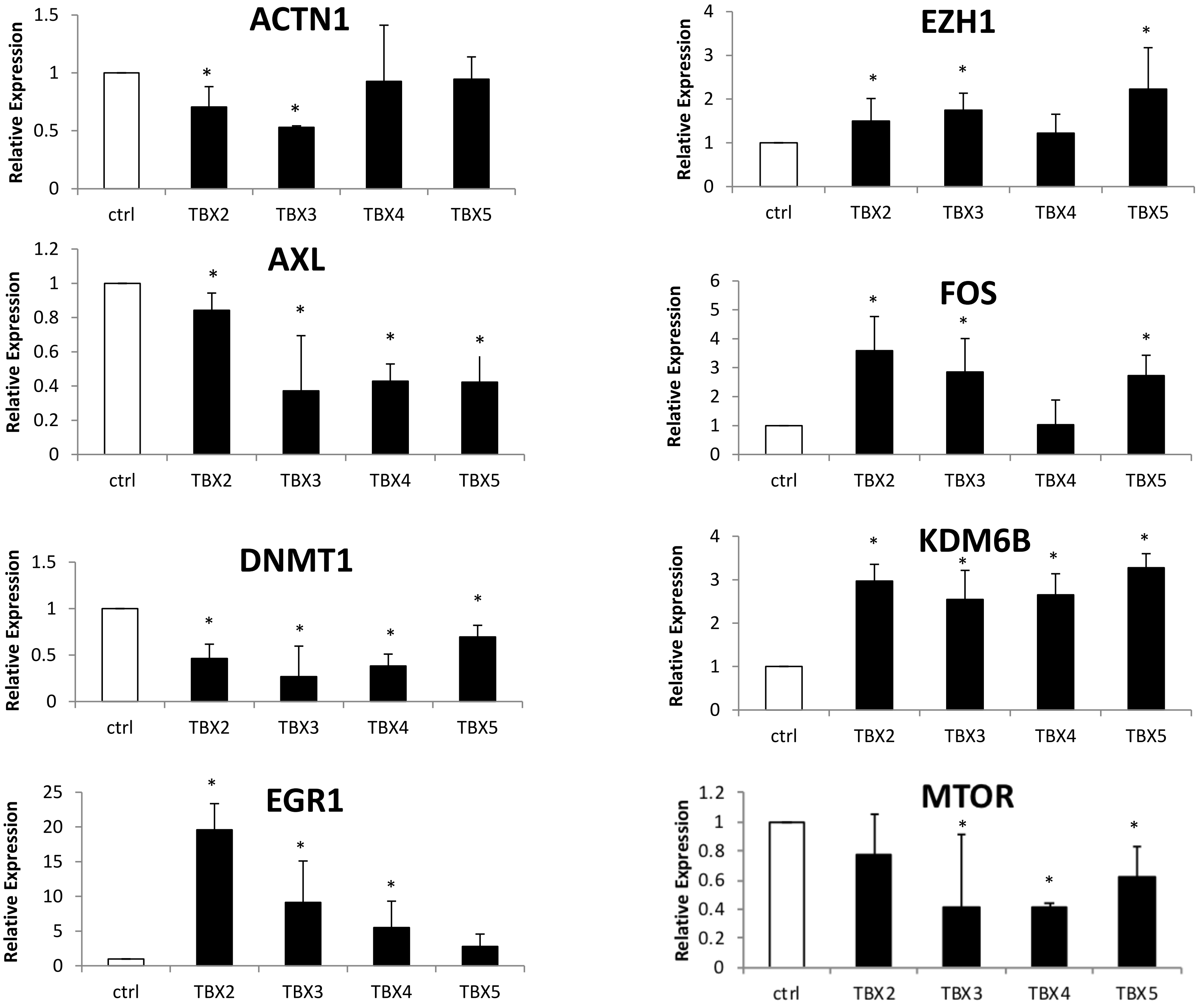

Supplement: Supplementary Figure 1 — Expression of TBX2 and 5 in adult human lung tissue. The αTBX2 stained slide shows nuclear and cytoplasmic staining (brown color) in bronchial epithelium (arrows in B) and alveolar lining (pneumocytes) with more prominent nuclear staining. While αTBX5 shows a staining within bronchial epithelium but it is less intense andlacks differential intensity between nucleus and cytoplasm; this is in contrast with the IgG negative control (A). Magnification X5 (A) and X20 (B). [file Data_Sheet_7.PDF]
