# Supplementary material for: Transcriptomic Alterations in Lung Adenocarcinoma Unveil New Mechanisms Targeted by the TBX2 Subfamily of Tumor Suppressor Genes
Source: Front Oncol. 2018 Oct 30;8:482. doi: 10.3389/fonc.2018.00482 (PMC6218583; doi:10.3389/fonc.2018.00482)
Supplement: Supplementary Table 1 — List of genes with their corresponding primers used in qPCR. [file Data_Sheet_1.PDF]

Supplementary Table 1: List of genes with their corresponding primers used in qPCR.

Gene name (Human)	Forward Primer	Reverse Primer
**ACTN1**	5' CGCATCATGAGCATTGTGGA 3'	5' GCAGCTCGTCCATGGTAATG 3'
**ANLN**	5' CTTAGTGCTGTGCGAACCAG 3'	5' CACACCATCTTCGATGCCAG 3'
**AXL**	5' GAGGGAGAGTTTGGAGCTGT 3'	5' GAAACAGACACCGATGAGCC 3'
**beta actin**	5' CATGGAGAAAATCTGGCACC 3'	5' TGATCTGGGTCATCTTCTCG 3'
**CCNG2**	5' GTGCCTACATGCTGCTCTTC 3'	5' AAGGAAAGCTGCCAGTCTCT 3'
**CDK1**	5' GGGGTCAGCTCGTTACTCAA 3'	5' TGACATGGGATGCTAGGCTT 3'
**DNMT1**	5' ACCAAGAACGGCATCCTGTA 3'	5' GCTGCCTTTGATGTAGTCGG 3'
**EGR1**	5' CTTCCCTTCCTCAGCTGTCA 3'	5' TAGAGAGGGAGGACTTGGCT 3'
**EIF3K**	5' CAAGTGCATGATCGACCAGG 3'	5' ATCCACACCTTTAGCTGGCT 3'
**EZH1**	5' TACCCTCAAGCTGGACTGTG 3'	5' TCTGCTCACTGAAGGAAGGG 3'
**EZH2**	5' TCGAGCTCCTCTGAAGCAAA 3'	5' AGTATCCACATCCTCAGCGG 3'
**FOS**	5' GCTTCAACGCAGACTACGAG 3'	5' AGTGACCGTGGGAATGAAGT 3'
**GAPDH**	GTCAGTGGTGGACCTGACCT	5' TCGCTGTTGAAGTCAGAGGA 3'
**HIST1H2BK**	5' ATTACAACAAGCGCTCGACC 3'	5' GGTTGGGCTTTAAGACGCTT 3'
**KDM1A**	5' GCCACACCTCTCTCAACTCT 3'	5' CCTGTCGCACTGCTGTATTC 3'
**KDM6B**	5' CTACTTGACGGGTTCCTGGT 3'	5' TCCACTCGTATCGTTCCAGG 3'
**MTOR**	5' CCTGCCTTTGTCATGCCTTT 3'	5' CTGGGTTTGGATCAGGGTCT 3'
**PRMT3**	5' TGTCAGAACCTGCTCGTCAT 3'	5' AGTTCCACACCCAACATCCA 3'
**TBX2**	5' TAAAGGAGGAGTGGGGTCCT 3'	5' CACGTAGACCACAGGGCAGT 3'
**TBX3**	5' TCCATGAGGGTGTTTGATGA 3'	5' ACCCTCGCTGGGACATAAAT 3'
**TBX4**	5' GCAGACCATCGAGAACATCA 3'	5' AGGGACAATGTCAATCAGCA 3'
**TBX5**	5' TCATAACCAAGGCTGGAAGG 3'	5' GCCCGTCACAGACCATTTAT 3'
**TET2**	5' ACAGAAGCAAGAACAGCAGC 3'	5' AGCTTGCAGGTGGATTCTCT 3'
