# Supplementary material for: Transcriptomic Alterations in Lung Adenocarcinoma Unveil New Mechanisms Targeted by the TBX2 Subfamily of Tumor Suppressor Genes
Source: Front Oncol. 2018 Oct 30;8:482. doi: 10.3389/fonc.2018.00482 (PMC6218583; doi:10.3389/fonc.2018.00482)
Supplement: Supplementary Table 2 — Common deregulated genes with their expression change and CGC annotation. [file Data_Sheet_2.PDF]

Supplementary Table S2: **Common deregulated genes with their expression change and CGC annotation**

Gene	Expression change	CGC annotation
ZNF436	up	
SESN2	up	
MTF1	up	
KDM4A	up	
ATP6V0B	up	
MYSM1	up	
TRMT13	up	
TAF13	up	
POU2F1	up	
SCYL3	up	
SUCO	up	
TOR1AIP2	up	
ZBTB41	up	
ARID4B	up	
PFKFB3	up	
CHST3	up	
HPS5	up	
RAB30	up	
GABARAPL1	up	
FGFR1OP2	up	
HELB	up	
THAP2	up	
CRY1	up	
TCTN2	up	
KLHL28	up	
TOGARAM1	up	
FOS	up	
SEL1L	up	
SECISBP2L	up	
FAM214A	up	
CHD2	up	tumor supressor
FBRS	up	
ZNF267	up	
TXNL4B	up	
OSGIN1	up	
MAP1LC3B	up	
KDM6B	up	
MBTD1	up	
ZNF317	up	
ZNF844	up	
ZNF430	up	
ZNF461	up	
ZNF568	up	
ZNF585A	up	
ZNF570	up	
ZNF222	up	
ZNF225	up	
ZNF235	up	
ZNF615	up	
ZNF432	up	
ZNF845	up	
ZNF264	up	
ZNF805	up	
ZIK1	up	
ZNF211	up	
KIDINS220	up	
CCDC121	up	
MXD1	up	
MFSD9	up	
CAB39	up	
JAG1	up	
SPATA2	up	
HSPA13	up	
POFUT2	up	
MAFF	up	
EAF1	up	
TMF1	up	
RYBP	up	
RPL32P3	up	
ZBTB49	up	
CPEB2	up	
CCNG2	up	
TET2	up	tumor suppressor
MYO10	up	
PARP8	up	
MTX3	up	
LUCAT1	up	

ARRDC3	up	
EGR1	up	
SLU7	up	
CREBRF	up	
BTN2A1	up	
ZKSCAN8	up	
HLA-L	up	
HLA-E	up	
DDR1	up	
DDX39B	up	
NEU1	up	
DNAH8	up	
ZNF451	up	
LCA5	up	
ZUFSP	up	
TNFAIP3	up	tumor supressor
SYNE1	up	
ZNF117	up	
ERV3-1-ZNF117	up	
APTR	up	
RBM48	up	
BUD31	up	
ZKSCAN5	up	
FAM200A	up	
PNPLA8	up	
CNOT4	up	
KDM7A	up	
YTHDF3	up	
SNX16	up	
KLF10	up	
RNF139-AS1	up	
ZCCHC6	up	
ZBTB43	up	
OFD1	up	
ATP7A	up	
B3GALT6	down	
MRPL20	down	
SSU72	down	
PARK7	down	
CTNNBIP1	down	
PGD	down	
MTOR	down	oncogene
CROCC	down	
RCC1	down	
EBNA1BP2	down	
SERBP1	down	
HIST2H2BE	down	
SHC1	down	
FDPS	down	
CSRP1	down	
NUCKS1	down	
HNRNPF	down	
CDK1	down	
PPA1	down	
MRPS16	down	
RPS24	down	
GLUD1	down	
MYOF	down	
MMS19	down	
OLMALINC	down	
BUB3	down	
POLR2L	down	
KIAA1549L	down	
FJX1	down	
MTCH2	down	
FADS1	down	
FIBP	down	

SORL1	down	
NTM	down	
EMP1	down	
TUBA1B	down	
TUBA1A	down	
EIF4B	down	
CBX5	down	
HNRNPA1	down	
PA2G4	down	
NAP1L1	down	
MMAB	down	
ACTN1	down	
TDP1	down	
CALM1	down	
CALM2	down	
CALM3	down	
DYNC1H1	down	
AHNAK2	down	
CDCA4	down	
THBS1	down	
EIF2AK4	down	
ANXA2	down	
KIF23	down	
NME4	down	
MRPS34	down	
TRAP1	down	
RPL23	down	
LASP1	down	oncogene
ACLY	down	
ITGA3	down	
KPNA2	down	
TRIM65	down	
TUBB6	down	
TMEM259	down	
LMNB2	down	
LDLR	down	
RAD23A	down	
PKN1	down	
UQCRFS1	down	
URI1	down	
PDCD5	down	
TDRD12	down	
GPI	down	
HAUS5	down	
COX6B1	down	
EIF3K	down	
ACTN4	down	
AKT2	down	oncogene
AXL	down	
HNRNPUL1	down	
PAFAH1B3	down	
TOMM40	down	
ERCC2	down	tumor supressor
ERCC1	down	
VASP	down	
OPA3	down	
SNRPD2	down	
CARD8	down	
EMP3	down	
PRMT1	down	
PXDN	down	
PPM1G	down	
CDC42EP3	down	
SEPT10	down	
ANAPC1	down	
TTL	down	
PKP4	down	

STK39	down	
TFPI	down	
BCL2L1	down	
AHCY	down	
CYP24A1	down	
SYCP2	down	
LSS	down	
SLC25A1	down	
NF2	down	tumor supressor
CARD10	down	
NUP210	down	
DCBLD2	down	
MGLL	down	
TM4SF18	down	
TM4SF1	down	
RSRC1	down	
KLHL5	down	
PLAC8	down	
INPP4B	down	
CFAP97	down	
RAI14	down	
OXCT1	down	
SH3TC2	down	
G3BP1	down	
NPM1	down	oncogene
ERGIC1	down	
RACK1	down	
HIST1H2BC	down	
HIST1H2AC	down	
HIST1H3D	down	
HIST1H1B	down	
HIST1H3J	down	
TRIM27	down	oncogene
CUL7	down	
EEF1A1	down	
ASCC3	down	
CRYBG1	down	
ARHGAP18	down	
ADGRG6	down	
UTRN	down	
ACAT2	down	
RAC1	down	oncogene
ANLN	down	
POLD2	down	
OGDH	down	
PPIA	down	
FDFT1	down	
LOXL2	down	
MLLT3	down	oncogene
GNE	down	
ANXA1	down	
C9orf40	down	
C9orf64	down	
FAM120A	down	
CTNNAL1	down	
KIAA0368	down	
FAM129B	down	
RPL7A	down	
EIF1AX	down	oncogene
LOC107984923	down	
SMS	down	
NONO	down	oncogene
BEX3	down	
RBMX	down	
