# Supplementary material for: Transcriptomic Alterations in Lung Adenocarcinoma Unveil New Mechanisms Targeted by the TBX2 Subfamily of Tumor Suppressor Genes
Source: Front Oncol. 2018 Oct 30;8:482. doi: 10.3389/fonc.2018.00482 (PMC6218583; doi:10.3389/fonc.2018.00482)
Supplement: Supplementary Table 3 — Common Regulated Genes in Reactome Pathways Deregulated in NCI-H1299 cells overexpressing TBXs. [file Data_Sheet_3.PDF]

Supplementary Table S3: Common Regulated Genes in Reactome Pathways Deregulated in NCI-H1299 cells overexpressing TBXs

Direction GSEA	Reactome Pathway	Genes Fold (red=up ; blue= down) & FDR < 0.1							
down	Activation of the mRNA upon binding of the cap-binding complex and eIFs	EIF1AX	EIF3K	EIF4B	RPS24				
down	Chromosome Maintenance	HIST1H2AC	HIST1H2BC	HIST2H2BE	NPM1	POLD2			
down	DNA Double-Strand Break Repair	ERCC1	HIST1H2BC	HIST2H2BE	KDM4A	KPNA2	POLD2	TDP1	
down	GTP hydrolysis and joining of the 60S ribosomal subunit	EIF1AX	EIF3K	EIF4B	RPL23	RPL7A	RPS24		
down	Gene Silencing by RNA	HIST1H2AC	HIST1H2BC	HIST2H2BE	NUP210	POLR2L	TDRD12		
down	Mitotic Metaphase and Anaphase	ANAPC1	BUB3	DYNC1H1					
down	Mitotic Prophase	CDK1	HIST1H2AC	HIST1H2BC	HIST2H2BE	NUP210			
down	Amyloid fiber formation	HIST1H2AC	HIST1H2BC	HIST2H2BE	SORL1				
down	Mitotic Prometaphase	BUB3	CDK1	DYNC1H1	HAUS5	OFD1	TUBA1A		
down	Cholesterol biosynthesis	ACAT2	FDFT1	FDPS	LSS				
down	Interleukin-12 signaling	ANXA2	HNRNPF	PPIA					
down	RMTs methylate histone arginines	HIST1H2AC	PRMT1						
down	Global Genome Nucleotide Excision Repair (GG-NER)	ERCC1	ERCC2	POLD2	RAD23A				
down	Formation of tubulin folding intermediates by CCT/TriC	TUBA1A	TUBA1B	TUBB6					
down	DNA strand elongation	POLD2							
down	HIV Infection	ERCC2	NPM1	NUP210	POLR2L	PPIA	RAC1	RCC1	TAF13
down	Signaling by ROBO receptors	RAC1	RPL23	RPL7A	RPS24	VASP			
down	RHO GTPases activate PKNs	HIST1H2AC	HIST1H2BC	HIST2H2BE	PKN1	RAC1			
down	Smooth Muscle Contraction	ANXA1	ANXA2	CALM1					
down	ERCC6 (CSB) and EHMT2 (G9a) positively regulate rRNA expression	HIST1H2AC	HIST1H2BC	HIST2H2BE					
down	TCF dependent signaling in response to WNT	AKT2	CTNNBIP1	HIST1H2AC	HIST1H2BC	HIST2H2BE			
down	Apoptosis	AKT2	BCL2L1	HIST1H1B					
down	Metabolism of non-coding RNA	NUP210	SNRPD2						
down	Base Excision Repair	POLD2							
up	HDMs demethylate histones	KDM4A	KDM6B	KDM7A					
