# Supplementary material for: Transcriptomic Alterations in Lung Adenocarcinoma Unveil New Mechanisms Targeted by the TBX2 Subfamily of Tumor Suppressor Genes
Source: Front Oncol. 2018 Oct 30;8:482. doi: 10.3389/fonc.2018.00482 (PMC6218583; doi:10.3389/fonc.2018.00482)
Supplement: Supplementary Table 4 — Enrichment scores and corresponding p-value from the 75 most up-regulated and down-regulated genes in TBXs with respect to ranked gene signatures from hypomethylating agents. [file Data_Sheet_4.PDF]

Supplementary Table S4: Enrichment scores and corresponding p-value from the 75 most up-regulated and down-regulated genes in TBXs with respect to ranked gene signatures from hypomethylating agents

ID	GENE	Enrichment.Score	p-value
GSE29077_azacitidine	TBX2	0.69597	7.82E-07
GSE29077_azacitidine	TBX3	0.61367	7.83E-07
GSE29077_azacitidine	TBX4	0.569595	7.96E-06
GSE29077_azacitidine	TBX5	0.464825	0.000935865567
GSE29077_decitabinet	TBX2	0.436445	0.0007151293753
GSE29077_decitabinet	TBX3	0.474945	0.0002227979506
GSE29077_decitabinet	TBX4	0.529965	7.08E-06
GSE29077_decitabinet	TBX5	0.47599	0.0001655164308
