# Supplementary material for: Transcriptomic Alterations in Lung Adenocarcinoma Unveil New Mechanisms Targeted by the TBX2 Subfamily of Tumor Suppressor Genes
Source: Front Oncol. 2018 Oct 30;8:482. doi: 10.3389/fonc.2018.00482 (PMC6218583; doi:10.3389/fonc.2018.00482)
Supplement: Supplementary Table 5 — Enrichment scores and corresponding p-value from the 75 most up-regulated and down-regulated genes in TBXs with respect to ranked gene signatures from public data in GEO. [file Data_Sheet_5.PDF]

Supplementary Table S5: Enrichment scores and corresponding p-value from the 75 most up-regulated and down-regulated genes in TBXs with respect to ranked gene signatures from public data in GEO

ID	GENE	Enrichment.Score	p-value
GSE10072	TBX2	-0.31086	0.2093140585
GSE10072	TBX3	-0.41011	0.006989404547
GSE10072	TBX4	0	1
GSE10072	TBX5	-0.38233	0.01787806028
GSE27262	TBX2	-0.249515	0.6766295164
GSE27262	TBX3	-0.427815	0.000275087743
GSE27262	TBX4	0	1
GSE27262	TBX5	-0.347115	0.03344658301
GSE43458	TBX2	-0.302855	0.03312991738
GSE43458	TBX3	-0.38495	0.000318143323
GSE43458	TBX4	0	1
GSE43458	TBX5	-0.31804	0.01314718379
