# Supplementary material for: Transcriptomic Alterations in Lung Adenocarcinoma Unveil New Mechanisms Targeted by the TBX2 Subfamily of Tumor Suppressor Genes
Source: Front Oncol. 2018 Oct 30;8:482. doi: 10.3389/fonc.2018.00482 (PMC6218583; doi:10.3389/fonc.2018.00482)
Supplement: Supplementary Table 6 — Enrichment scores and corresponding p-value from the 75 most up-regulated and down-regulated genes in TBXs with respect to ranked gene signatures from a murine tumor model dataset. [file Data_Sheet_6.PDF]

Supplementary Table S6: Enrichment scores and corresponding p-value from the 75 most up-regulated and down-regulated genes in TBXs with respect to ranked gene signatures from a murine tumor model dataset

ID	GENE	Enrichment.Score	p-value
ADCvNormal	TBX2	-0.43582	0.07938263799
ADCvNormal	TBX3	-0.31924	0.227146148
ADCvNormal	TBX4	0	1
ADCvNormal	TBX5	-0.389725	0.1706007767
ADNvNormal	TBX2	0	1
ADNvNormal	TBX3	0	1
ADNvNormal	TBX4	0	1
ADNvNormal	TBX5	0	1
TumorvNormal	TBX2	0	1
TumorvNormal	TBX3	-0.288555	0.5396252403
TumorvNormal	TBX4	0	1
TumorvNormal	TBX5	0	1
